# Infection by a helminth parasite is associated with changes in DNA methylation in the house sparrow

**DOI:** 10.1002/ece3.9539

**Published:** 2022-11-27

**Authors:** Sarah L. Lundregan, Hannu Mäkinen, Amberly Buer, Håkon Holand, Henrik Jensen, Arild Husby

**Affiliations:** ^1^ Department of Biology, Centre for Biodiversity Dynamics Norwegian University of Science and Technology Trondheim Norway; ^2^ Evolutionary Biology, Department of Ecology and Genetics Uppsala University Uppsala Sweden

**Keywords:** DNA methylation, epigenetics, immunity, parasite, passerine, RRBS

## Abstract

Parasites can exert strong selective pressures on their hosts and influence the evolution of host immunity. While several studies have examined the genetic basis for parasite resistance, the role of epigenetics in the immune response to parasites is less understood. Yet, epigenetic modifications, such as changes in DNA methylation, may allow species to respond rapidly to parasite prevalence or virulence. To test the role of DNA methylation in relation to parasite infection, we examined genome‐wide DNA methylation before and during infection by a parasitic nematode, *Syngamus trachea*, in a natural population of house sparrows (*Passer domesticus*) using reduced representation bisulfite sequencing (RRBS). We found that DNA methylation levels were slightly lower in infected house sparrows, and we identified candidate genes relating to the initial immune response, activation of innate and adaptive immunity, and mucus membrane functional integrity that were differentially methylated between infected and control birds. Subsequently, we used methylation‐sensitive high‐resolution melting (MS‐HRM) analyses to verify the relationship between methylation proportion and *S. trachea* infection status at two candidate genes in a larger sample dataset. We found that methylation level at *NR1D1*, but not *CLDN22*, remained related to infection status and that juvenile recruitment probability was positively related to methylation level at *NR1D1*. This underscores the importance of performing follow‐up studies on candidate genes. Our findings demonstrate that plasticity in the immune response to parasites can be epigenetically mediated and highlight the potential for epigenetic studies in natural populations to provide further mechanistic insight into host–parasite interactions.

## Graphical abstract

Parasites can exert strong selective pressures on their hosts and DNA methylation change may allow species to respond rapidly to parasite infection. We examined genome‐wide DNA methylation before and during infection of house sparrows by a parasitic nematode and found that DNA methylation levels are slightly lower in infected sparrows and identified candidate genes relating to initial immune response, activation of innate and adaptive immunity, and mucus membrane functional integrity. Then, we used methylation‐sensitive high‐resolution melting to verify that methylation level at NR1D1, but not CLDN22, remained related to infection status in a larger sample dataset, and that juvenile recruitment probability was positively related to methylation level at NR1D1. Thus, our findings demonstrate that plasticity in the immune response to parasites can be epigenetically mediated.

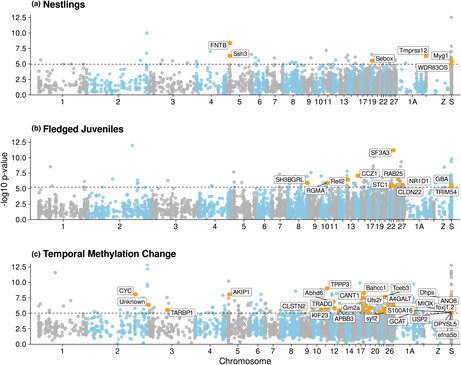

## 1 INTRODUCTION

Parasites are important drivers of natural selection and can have major effects on individual fitness, population dynamics, and evolutionary processes in natural populations (Morgan et al., 2004). Complex changes in parasite prevalence and virulence that may have negative consequences for species communities are expected in the current period of rapid environmental change (Altizer et al., 2013). Resistance to parasites can have a genetic basis involving numerous genetic pathways that shape total immune response (Allen & Maizels, 2011; Hagai et al., 2018). Transcriptional regulation of immune genes has a pivotal role in mounting appropriate immune response to pathogens, through mechanisms including regulatory action of non‐coding RNAs, transcriptional control of B and T Cell development (Rothenberg, 2014), and changes in chromatin structure that contribute to regulation of both innate and adaptive immunity (Smale et al., 2014). Infection by parasites is known to alter expression of immune genes, which has been well‐studied in model organisms (Maizels et al., 2018) and in livestock, including sheep (Andronicos et al., 2010; Pemberton et al., 2011), cattle (Li et al., 2011), and chicken (Dalgaard et al., 2015). However, the factors that modulate these changes in gene expression remain largely unknown. Thus, investigating epigenetic mechanisms that can modulate regulation of immune genes is central to understanding the molecular basis of the immune response to parasite infection.

DNA methylation is the most well‐studied form of epigenetic modification, and there is mounting evidence that DNA methylation variation at loci involved in both innate and adaptive immunity are integral to the immune response to pathogens (Kondilis‐Mangum & Wade, 2013; Saeed et al., 2014; Weng et al., 2012; Zhang & Cao, 2019). DNA methylation involves the addition of a methyl (‐CH3) group on the 5′ carbon of a cytosine residue by DNA methyltransferase. In vertebrates, methylation predominantly occurs at CG (CpG) dinucleotides but may also occur at non‐CpG sites (Auclair & Weber, 2012). Cytosine methylation at a promoter or transcription start site (TSS) generally reduces gene expression via inhibition of transcription factor binding or reduction of transcription rate (Deaton & Bird, 2011), whereas CpG methylation in other genomic contexts may serve a regulatory role or promote expression of non‐coding RNAs (Auclair & Weber, 2012). In birds, gene body methylation does not appear to influence gene expression levels in blood (Watson et al., 2020), but may influence expression in other tissues, notably brain tissue (Derks et al., 2016).

The environmentally responsive nature of DNA methylation (Angers et al., 2010) provides a mechanism for rapid response of host's immune systems to environmental pathogens, the effects of which may be either transient or trans‐generational (Poulin & Thomas, 2008; Roth et al., 2018). However, relatively few studies to date have investigated changes in DNA methylation in response to parasite infection. In mice, DNA methylation has been shown to play a central role in dendritic cell‐induced Th2 immunity to helminth parasites, via regulation of key target genes (Cook et al., 2015). Parasite‐mediated selection has also been found to modulate DNA methylation in natural populations of killifish (*Kryptolebias hermaphroditus*; Berbel‐Filho et al., 2019). In Trinidadian guppies (*Poecilia reticulata*) experimentally infected by an ectoparasitic monogenean, the authors identified differentially methylated regions between infected and control fish that overlapped genomic regions relevant to immune response (Hu et al., 2018). In three‐spined stickleback (*Gasterosteus aculeatus*) experimentally infected by a parasitic nematode, genome‐wide changes in DNA methylation have been observed (Sagonas et al., 2020). A study on DNA methylation in a natural population of red grouse (*Lagopus lagopus scotica*) also detected an epigenetic signature of parasite infection in epilocus‐specific differentiation at immune genes and sites relating to histone acetylation, whereas genome‐wide changes in methylation levels related to parasite load were not present (Wenzel & Piertney, 2014). Conversely, experimental manipulation of parasite presence in mockingbirds (*Mimus parvulus*) did not result in a strong epigenetic signature of infection (McNew et al., 2021). Parasites may also modulate the immune response of their hosts by inducing DNA methylation changes that dampen the inflammatory response to infection (Varyani et al., 2017; Zakeri et al., 2018).

The house sparrow (*Passer domesticus*, Linnaeus, 1758) is a passerine bird with worldwide distribution that has been used as an ecological model species in several genetic architecture studies on morphological (Jensen et al., 2003; Lundregan et al., 2018; Silva et al., 2017), physiological (Andrew et al., 2018, 2019), and parasite resistance traits (Lundregan et al., 2020). Individual‐level data on infection by a parasitic nematode, *Syngamus trachea* (Montagu, 1811), have been systematically collected by feces sampling in our study system of a metapopulation of house sparrows in northern Norway (Holand et al., 2013, 2019). *Syngamus trachea* is circumglobally distributed and infects most terrestrial bird genera (Atkinson et al., 2008; Campbell, 1935). Symptoms of infection range from mild to severe and include gasping, reduced food intake, and anemia, which may lead to increased mortality (Atkinson et al., 2008). Thus, infection by this parasite may affect ecological processes by curtailing wild bird populations. *Syngamus trachea* eggs are excreted in the feces of infected birds and transmission occurs by ingestion of mature larvae present in the environment or by ingestion of paratenic invertebrate hosts, usually earthworms (Barus, 1966a). Once ingested, larvae migrate to the lungs via the vascular system and mature nematodes are found in the trachea of infected birds (Atkinson et al., 2008). The prepatent period in chickens (*Gallus gallus*) is approximately 12 days, and the first parasite eggs are found in host feces approximately 15 days after infection (Fernando et al., 1971). Thus, house sparrow nestlings are less likely to be infected by *S. trachea* because they do not commonly ingest earthworms that are the major paratenic host, and any birds that are infected in the nest are in the prepatent stage of infection prior to fledging, which usually occurs at an age of 14–16 days (Anderson, 2006).

Innate immunity is important for defense against nematode parasites in vertebrates (De Veer et al., 2006), and increased numbers of pro‐inflammatory cytokines have been measured in chicken during nematode infection (Dalgaard et al., 2015). Adaptive immunity has also been shown to be important in the defense against parasitic nematodes in chicken (Andersen et al., 2013), and several poultry species have been shown to mount an adaptive immune response to infection by *S. trachea* (Olivier, 1944). Furthermore, *S. trachea* has been found to secrete proteolytic enzymes that act as immunomodulatory agents and interfere with immune signaling pathways (Riga et al., 1995), which could lead to downstream epigenetic alterations in infected house sparrows. *Syngamus trachea* prevalence varies spatiotemporally within our study system (Holand et al., 2013), is positively associated with temperature, and is higher following mild winters (Holand et al., 2019). Infection by the parasite reduces survival probability in juvenile and adult house sparrows (Holand et al., 2014) and negatively influences female reproductive success (Holand et al., 2015). Resistance to *S. trachea* has been shown to be polygenic in nature in the house sparrow and has been associated with several genes involved in innate and adaptive immune function, with genes linked to mucus membrane integrity and ciliogenesis, and with genes involved in physiological processes such as production of reactive oxygen species and vitamin A synthesis (Lundregan et al., 2020). The well‐documented effects of *S. trachea* infection in this metapopulation of house sparrows, alongside genomic resources in the form of an annotated genome (Elgvin et al., 2017), make the house sparrow‐nematode system a particularly well‐suited study system for further research into possible epigenetic drivers of resistance to parasites in natural populations.

In this study, we use reduced representation bisulfite sequencing (RRBS) to assess whether infection by *S. trachea* alters genome‐wide DNA methylation levels, as well as site‐specific patterns of DNA methylation in house sparrows. We used a repeated sampling design to sample individuals while still in the nest (henceforth referred to as the “nestling stage”) when they were assumed to be uninfected, and again after fledging (henceforth the “fledged juvenile stage”) when individuals in our “case” group were infected by *S. trachea*. This was done to help us understand the causality of any DNA methylation differences between case and control birds: do epigenetic differences at the nestling stage when all birds are uninfected influence later probability of infection, or does infection result in subsequent DNA methylation changes? First, we explored whether genome‐wide DNA methylation profiles were different between case and control birds using redundancy analyses (RDA). Next, we used differential methylation analysis to determine whether any parasite‐induced changes in DNA methylation were associated with genes relating to immune function, physical expulsion of parasites (for example by influencing mucus production, ciliary function, or mucus membrane integrity) or physiological processes relating to immunity. Then, we used gene ontology (GO) analysis to determine whether any of the CpG sites associated with parasite infection status were enriched for functional groups. Finally, we used methylation‐sensitive high‐resolution melting (MS‐HRM) analyses to validate the relationship between methylation levels at two of our candidate genes and *S. trachea* infection status in a larger sample dataset.

## 2 MATERIALS AND METHODS

### 2.1 Sampling and experimental design

The individuals used in this study were sampled as part of our long‐term study of an insular house sparrow metapopulation off the coast of northern Norway (66°30′N, 12°30′E). The RRBS dataset included 12 female birds that were nestlings and fledged juveniles on Hestmannøy in 2011, and 10 female birds that were adults on one of three islands in the metapopulation (Hestmannøy, Gjerøy, or Aldra) between 2008 and 2011 (Table 1). The MS‐HRM dataset included 322 fledged juvenile house sparrows that were sampled on one of five islands in the metapopulation (Hestmannøy, Gjerøy, Aldra, Træna, or Indre Kvarøy) between 2009 and 2013 (Table 2). See the Materials and Methods section “Candidate gene verification using MS‐HRM” for details of MS‐HRM analyses on the relationship between methylation proportion in the promoter regions of our candidate genes and *S. trachea* infection status. Data collection was carried out under permission from the animal experimentation administration (FOTS) of the Norwegian Food Safety Authority (NFSA) and in accordance with permits from the Norwegian Bird Ringing Centre. All birds were ringed with a unique combination of three colored plastic rings as well as a metal ring with an individual identification number, either as nestlings in the nest or upon first capture by mist netting. A 25 μl blood sample was collected from the brachial vein for nestlings in the nest, and for fledged juveniles and adults upon each capture occasion. Blood samples were stored in 96% ethanol at −20°C. Blood is the most relevant tissue for studying DNA methylation changes in response to pathogens because it contains the white blood cells that orchestrate the immune response, as well as other molecules involved in immunity. However, see the “Caveats and future directions” section for a discussion of the limitations of using whole blood. Fledged juveniles and adults were sampled for feces on each capture occasion, birds were placed in a paper bag for approximately 15 min prior to blood sample collection to allow time for them to produce a feces sample. Fecal samples were collected and stored in ~1 ml of MilliQ water at 4°C until processed. *Syngamus trachea* fecal egg count (FEC) was quantified using the sucrose flotation method described in Holand et al. (2013). Island‐specific microsatellite pedigrees are available for all birds used in this study and were used to estimate the relatedness between individuals (Araya‐Ajoy et al., 2019; Billing et al., 2012).

**TABLE 1:** RRBS sampling design showing individuals available for methylation analyses in the juvenile and adult datasets

ID	Island	Experimental group	Sample time point 1		FEC	Sample time point 2		FEC
Juvenile dataset								
8N06323	Hestmannøy	Case	2011/06/05	Nestling	‐	2011/07/20	Fledged juvenile	262
8N06545			2011/06/25		‐	2011/07/20		10
8M71818			2011/07/01		‐	2011/07/24		10
8M71882			2011/07/03		‐	2011/07/20		13
8N06359			2011/06/05		‐	2011/07/01		57
8N06539			2011/06/25		‐	2011/07/17		96
8N06587		Control	2011/06/30		‐	2011/07/17		0
8N06958			2011/07/29		‐	2011/08/13		0
8M71821			2011/06/30		‐	2011/07/20		0
8M71823			2011/07/03		‐	2011/07/17		0
8M71828			2011/07/03		‐	2011/07/20		0
8M71819			2011/06/30		‐	2011/07/17		0
Adult dataset								
8309263	Hestmannøy	Case	2008/07/20	Infected	12	‐		
8816918	Hestmannøy		2009/10/07		4			
8L26591	Hestmannøy		2010/08/14		6			
8M31507	Gjerøy		2009/10/05		37			
8M71205	Hestmannøy		2010/10/03		94			
8N06560	Gjerøy	Control	2012/10/11	Never infected	0			
8L89503	Hestmannøy		2010/07/21		0			
8L19928	Aldra		2008/10/03		0			
8M72863	Gjerøy		2011/05/19		0			
8L89516	Hestmannøy		2010/10/09		0			

*Note*: In the juvenile dataset, cases are birds infected by *Syngamus trachea* (defined as birds with a fecal egg count (FEC) > 0, *n* = 6 individuals) on the day the fledged juvenile blood sample was collected (sample time point 2), controls are birds that were never infected by the parasite as juveniles (*n* = 6 individuals). In the adult dataset, cases are birds infected by *S. trachea* (FEC > 0) on the day of blood sampling (*n* = 5 individuals), controls are birds that never had FEC > 0 and were sampled for feces several times throughout their lifespan (*n* = 5 individuals).

**TABLE 2:** Sampling design for MS‐HRM analyses, showing the number of individuals from each study island for which samples were successfully amplified during the PCR stage for each gene (*NR1D1*, *CLDN22*), as well as the number and proportion of infected individuals and totals.

Gene	Island	Num. individuals	Num. infected	Proportion infected
NR1D1	Træna	80	58	0.725
	Gjerøy	59	25	0.424
	Hestmannøy	96	68	0.708
	Indre Kvarøy	59	37	0.627
	Aldra	28	16	0.571
	TOTAL	322	204	0.634
CLDN22	Træna	77	56	0.727
	Gjerøy	55	21	0.382
	Hestmannøy	92	64	0.696
	Indre Kvarøy	57	35	0.614
	Aldra	27	16	0.592
	TOTAL	308	192	0.623

*Note*: All fledged juveniles that were infected by *Syngamus trachea* at the time of sampling (those with FEC greater than 0) on these five islands between 2009 and 2012 were included in the MS‐HRM analyses. Uninfected‐fledged juveniles (those with FEC equal to 0) were randomly selected among all uninfected‐fledged juveniles from the same islands and years.

Influence of parasite infection on DNA methylation patterns was investigated in juvenile birds using samples from 12 female house sparrows (Table 1), six of which were infected by *S. trachea* in their juvenile year (cases, individuals that had a FEC greater than zero when sampled at the fledged juvenile stage) and six of which were not infected as juveniles (controls, birds that were feces sampled multiple times during their juvenile year and never had a FEC greater than zero). Each bird was blood sampled twice: once at the nestling stage (10–14 days old) and later at the fledged juvenile stage when approximately 1‐month‐old (range: 26–37 days old, except for one individual that was sampled for the second time at 55 days old). This sampling design was chosen to help unravel the causality of any DNA methylation differences between cases and controls by allowing us to make inferences about whether differences in DNA methylation levels prior to infection by *S. trachea* (at the nestling stage) influence immune defense and susceptibility to infection, or whether infection (in cases at the fledged juvenile stage) could instead cause DNA methylation levels to change. A separate adult dataset was used to determine whether sites that showed at least 15% methylation difference between cases and controls in fledged juveniles were also differently methylated according to infection status in adult birds. This was done to broaden our understanding of whether any DNA methylation changes during infection by *S. trachea* were consistent between different life stages. The adult dataset consisted of 10 female house sparrows (Table 1), five of which were infected by *S. trachea* as adults and were blood and feces sampled during infection (cases), and five birds that were feces sampled at least once as juveniles and were never recorded as infected by *S. trachea* during their lifetime (controls). Differential methylation analysis was not carried out on the adult dataset because low rates of infection among adult house sparrows (Holand et al., 2013) led to sampling of adult birds from several island‐year combinations (Table 1), and the power to control for any spatio‐temporal variation in a generalized linear mixed model (GLMM) context was limited.

### 2.2 Sample processing

Total genomic DNA (gDNA) was extracted from whole blood samples preserved in 96% ethanol. Blood samples were first lysed by incubation in Lairds buffer with 90 μg of proteinase K for 3 h at 50°C. Automated extraction of gDNA from the lysate was carried out on a Biomek NX^p^ robot (Beckman Coulter) using the ReliaPrep Large Volume HT gDNA Isolation System (Promega). gDNA was eluted in nuclease‐free water and quality was assessed using nanodrop and by 1% agarose gel electrophoresis. Library preparation and subsequent sequencing were carried out at the Roy J. Carver Biotechnology Center, University of Illinois at Urbana‐Champaign, USA. Libraries were prepared using the Ovation RRBS Methyl‐Seq library preparation kit (Tecan), with approximately 1 μg of total genomic DNA for each sample. Library preparation steps comprised of DNA digestion with MspI to produce CCGG overhangs, bisulfite treatment, barcoded adapter ligation, and PCR amplification. The libraries were size‐selected for insert sizes of 20–200 base pairs (bp) and quantified by qPCR. Pools were then randomized across lanes before sequencing on a NovaSeq 6000 using single‐end, directional sequencing. Lanes were spiked with PhiX to improve nucleotide diversity.

### 2.3 Sequence alignment and identification of CpG sites

Prior to alignment of the RRBS sequence data, low‐quality bases and adaptor contamination were trimmed using TrimGalore! 0.6.7 with default parameters. Subsequently, trimming of diversity adapters was done using the “trimRRBSdiversityAdaptCustomers.py” Python script provided by NuGEN (v 2.3). Trimmed sequencing reads were aligned against an in‐silico RRBS digested, bisulfite‐converted version of the *P. domesticus* reference genome v 1.0 (Elgvin et al., 2017) using BiSulfite Bolt aligner (Farrell et al., 2021) in single‐end mode with default alignment parameters. Methylation bias plots (Hansen et al., 2012) for each sequenced individual were produced using MethylDackel v 0.3.0 to assess methylation percentage in each position of sequence reads, no trimming was performed before downstream analysis (Table A1 in Appendix 1). CpG sites were then identified for each sample using the “CallMethylation” function in BiSulfite Bolt. The R package Methylkit (Akalin et al., 2012) was used to determine read depth of CpG sites, and only sites with a minimum of 10x coverage (including methylated and unmethylated counts) that were identified across all samples in each downstream analysis were included. The final juvenile dataset consisted of 337,524 CpG sites that were shared between all 24 samples from nestlings and fledged juveniles (Table 1 and Table A2 in Appendix 1), and the adult dataset consisted of 553,161 CpG sites shared between all 10 adult individuals (Table 1 and Table A2 in Appendix 1). There were more 10× sites in the adult dataset because there were fewer samples, so each CpG site had a greater chance of having 10× coverage for all samples. In juveniles, we calculated mean methylation level across shared CpG sites for cases and controls at both the nestling and fledged juvenile stage. We then examined methylation patterns at these sites in more detail using redundancy analyses implemented in the R package Vegan (Borcard et al., 2011). The distance matrix used as the response matrix in each RDA was a site x individual matrix of percent methylation values. First, RDA was performed separately for nestling and fledged juvenile samples to determine whether infection status (case–control group, fitted as a fixed factor in the model) contributed more to genome‐wide variation in methylation profiles at the nestling stage when all individuals were either uninfected or at a very early stage of infection, or at the fledged juvenile stage when cases were infected by *S. trachea*. This approach made use of our repeated sampling design to help clarify the causality in any DNA methylation differences between cases and controls. Subsequently, RDA was performed on all samples together (including repeated measures for individuals at both the nestling and fledged juvenile stage, with both infection status and stage fitted as fixed factors in the model) to determine whether infection status or stage contributed more to variation in methylation profiles. To better understand whether the epigenetic response to parasite infection was the same at different life stages, we first calculated mean methylation levels for adult cases and controls using 5455 shared 10× sites that had at least 15% methylation difference between cases and controls in fledged juveniles. RDA was also performed on adult samples, using methylation levels at the same 5455 CpG sites as a response and case–control group as a fixed factor in the model, to determine whether infection status explained a significant proportion of the variance in methylation levels at these sites in adult house sparrows.

### 2.4 Influence of parasite infection on site‐specific DNA methylation

Differential methylation analyses using the RRBS data were carried out for juvenile birds using only CpG sites with at least 15% difference in mean methylation between cases and controls, resulting in 393,138 sites for the nestling dataset, and 619,626 sites for the fledged juvenile dataset. This filtering was used to prevent large deviations from a uniform *p*‐value distribution that arise due to inclusion of many sites that are not expected to show a difference in mean methylation between study groups (see Husby, 2022). GLMMs with binomial error distribution were implemented using the R package lme4qtl (Ziyatdinov et al., 2018), to identify differentially methylated cytosines (DMCs) while accounting for relatedness between individuals and population structure (Lea et al., 2017). Pedigree relatedness between individuals was calculated using the R package NADIV (Wolak, 2012). Differential methylation between cases and controls at both the nestling and fledged juvenile stage (4530 CpG sites were analyzed for nestlings, and 8834 sites for fledged juveniles) was assessed using

(1) $$y=\mu +x_{G}\beta_{G}+Z_{r}$$

where *y* is a two‐column matrix of methylated and unmethylated counts, *μ* is the intercept term, *x*_G_ is a vector relating individuals to case–control group, *β*_G_ is the group effect, and *Z*_r_ is the pedigree relationship matrix. Bonferroni correction of group effect *p*‐values with a family‐wise error rate (FWER) of .05 was used to identify CpG sites that were differentially methylated between cases and controls.

To examine whether temporal change in mean methylation level between the nestling and fledged juvenile stage differed between cases and controls (8921 analyzed CpG sites), a model including interaction between case–control group and stage was fit using

(2) $$y=\mu +x_{G}{\beta_{G}}^{*}\;x_{T}\beta_{T}+Z_{\mathrm{ID}}+Z_{r}$$

where *y* is a two‐column matrix of methylated and unmethylated counts, *μ* is the intercept term, *x*_G_ is a vector relating individuals to case–control group, *β*_G_ is the group effect, *x*_T_ is a vector relating samples to stage (nestling or fledged juvenile), *β*_T_ is the effect of stage, *Z*_ID_ is the random effect for individual repeated measurements, and *Z*_r_ is the pedigree relationship matrix. Bonferroni correction of interaction effect *p*‐values with FWER .05 was used to identify CpG sites for which there was evidence that the change in mean methylation level between the nestling and fledged juvenile stage was different between cases and controls.

### 2.5 Genomic locations and functional analysis

CpG sites in the RRBS dataset were assigned to their genomic location using an annotated version of the house sparrow genome (unpublished, from Elgvin et al., 2017). The R packages “GenomicFeatures” (Lawrence et al., 2013) and “rtracklayer” (Lawrence et al., 2009) were used to extract start and end positions for exons, first introns, other introns, transcription start sites (TSS, defined as the region 300 bp upstream to 50 bp downstream of the annotated gene start), and promoters (defined as the region 3 Kbp to 301 bp upstream of the annotated gene start). BEDtools v2.29.2 (Quinlan & Hall, 2010) was then used to define overlap between analyzed CpG sites and the specified genomic features (intergenic regions, promoters, TSS, exons, first introns, or other introns) and assign sites to these genomic features (Table A3 in Appendix 1). This annotation was used to assign the DMCs that were identified using differential methylation analyses to genes for functional gene ontology (GO) analysis. If DMCs were assigned to more than one genomic feature, for example to the promoter of one gene and the TSS of another gene, both assignments were included. Only DMCs in the TSS, promoter, or first intron of genes were used for functional analyses because the effects of methylation level in these genomic features are known (Anastasiadi et al., 2018; Auclair & Weber, 2012; Deaton & Bird, 2011). Functional analysis was done using the Cytoscape plugin ClueGO 2.5.8 (Bindea et al., 2009) using Bonferroni step‐down correction with a cutoff *p*‐value of .05, default Kappa score, and default network specificity. We used human (accessed 13.05.2021) as well as chicken (accessed 16.11.2021) gene ontologies, and terms from GO Biological Process and KEGG were considered, as well as Reactome Pathways when using human gene ontologies.

### 2.6 Candidate gene verification using MS‐HRM

#### 2.6.1 Gene regions and primer design

In differential methylation analyses with limited sample size, false‐positive DMCs are likely to be common and caution should be taken when constructing a narrative around putative candidate genes (Pavlidis et al., 2012). To address this issue, we used MS‐HRM analyses to verify the influence of *S. trachea* infection status on DNA methylation proportion at the candidate genes we identified at the fledged juvenile stage. All fledged juveniles that were infected by *S. trachea* at the time of sampling (those with FEC greater than 0) on five islands within the house sparrow metapopulation between 2009 and 2012 were included in the MS‐HRM analyses. Uninfected‐fledged juveniles (those with FEC equal to 0) that were sampled on the same islands during the same period were then randomly selected to total 350 individuals. All eight genes that had a DMC within the TSS or promoter at the fledged juvenile stage were considered for MS‐HRM analyses (See Table A4 in Appendix 1). Based on our selection criteria for CpG sites included in differential methylation analyses in the RRBS datasets (sites with at least 15% methylation difference between cases and controls), a large difference in methylation was anticipated between infected and uninfected individuals. Therefore, we used bisulfite sequencing primers (BSP) that did not contain CpG sites within the primer sequence to perform the MS‐HRM analyses because BSP primers are better suited to detecting large methylation differences than primers that contain CpG sites within the primer sequence (Life Technologies, 2010). Our BSP primers were designed using Methyl Primer Express v1.0 (Thermo Fisher Scientific) and the *P. domesticus* reference genome (Elgvin et al., 2017). Best practices for BSP primer design (Life Technologies, 2012) meant that high‐quality BSP primers could only be designed for four candidate genes with favorable distribution of other CpG sites near the DMC of interest (*NR1D1*, *CLDN22*, *Rell2*, and *SF3A3*). Where possible, BSP primers that amplified the DMC of interest for a candidate gene were designed; otherwise, we designed BSP primers that amplified a region less than 500 bp from the DMC of interest. Due to high spatial autocorrelation of DNA methylation in CpG regions within a few hundred bp (Eckhardt et al., 2006; Lea et al., 2017), the latter method was unlikely to impact the chance of detecting any extant relationship between methylation proportion at a given candidate gene and infection status.

#### 2.6.2 Methylated standards

Standard DNA is required in MS‐HRM to calculate the methylation proportion of the amplified regions. A 0% methylated and 100% methylated standard were created using a pool of 10 DNA samples from a randomized set of house sparrow individuals from the house sparrow metapopulation that were not included in the main MS‐HRM analyses. The 0% methylated standard was produced by performing whole genome amplification on the pooled DNA using the Illustra GenomiPhi V2 DNA Amplification Kit (Cytivia). The 100% methylated standard was produced by fully methylating the same pooled DNA using CpG methyltransferase (M.Sssl, Thermo Fisher Scientific). After quantification using the NanoDrop One Spectrophotometer (Thermo Fisher Scientific), standard DNA (0% and 100%) was purified using the GeneJet PCR Purification Kit (Thermo Fisher Scientific).

#### 2.6.3 Bisulfite conversion

gDNA was extracted from house sparrow whole blood samples using the same method as for RRBS sequencing and stored at −20°C until processing. gDNA samples and purified standard DNA were bisulfite converted using the EpiJET Bisulfite Conversion Kit (Thermo Fisher Scientific). The concentration of the bisulfite‐converted DNA samples was then measured using the NanoDrop One Spectrophotometer and adjusted to 20 ng/μl.

#### 2.6.4 MS‐HRM

Bisulfite‐converted DNA samples were PCR amplified followed by melt analysis using the QuantStudio 12 K Flex Real‐Time PCR System (Thermo Fisher Scientific) equipped with the High‐Resolution Melt Software for QuantStudio™ 12 K Flex Real‐Time PCR System (Applied Biosystems). Each sample was processed in duplicate. The HRM reactions were set up according to the MeltDoctor® HRM Master Mix Protocol (Applied Biosystems). The total volume of each reaction was 20 μl and contained 10 μl of MeltDoctor® HRM Master Mix, 1.2 μl of each 5 μM primer, 1 μl of 20 ng/μl template DNA, and 6.6 μl of deionized H_2_O. The PCR conditions for the two candidate gene regions that were successfully amplified, *NR1D1* and *CLDN22*, are presented in Table A6 in Appendix 1. Following PCR amplification, samples were denatured at 95°C for 10 s, then annealed at 60°C for 1 min, then heated to 95°C for 15 s at 1.6°C/s for gradual melting, and finally annealed at 60°C for 15 s. In total, samples from 322 individuals were successfully amplified for *NR1D1*, and samples from 308 individuals were successfully amplified for *CLDN22* (Table 2). Standard samples with known methylation levels (0%, 5%, 10%, 30%, 60%, and 100% for *NR1D1*, and 0%, 2.5%, 5%, 10%, 25%, and 100% for *CLDN22*) were included in duplicate on each plate and were later used to produce the standard curves used to estimate the methylation proportion for each sample. These standard series were prepared by combining 0% methylated and 100% methylated standard DNA in the appropriate ratios to approximate DNA samples with the same methylation level. Melting curves were automatically normalized by the high‐resolution melt software, relative to the pre‐melt and post‐melt temperature regions. For *NR1D1* the pre‐melt and post‐melt regions were set to 60.3–64.8 and 86.5–92.8°C respectively (dependent on plate), and for *CLDN22* the pre‐melt and post‐melt regions were set to 61.4–69.7 and 86.6–92.8°C, respectively. The 0% methylated standard was then set as a baseline to derive the difference curves from the first derivative of the melting curves (Hamano et al., 2016), and the “Df value” was defined as the maximum absolute value of the relative fluorescence signal differences for each sample.

#### 2.6.5 Data analysis

The Df values for the methylated standard series were used to create a standard curve for each plate. These standard curves follow the non‐linear regression model described in Hamano et al. (2016).

(3) $$\frac{a*M}{100-M}=\frac{\mathrm{Df}}{{\mathrm{Df}}_{\max}-\mathrm{Df}}$$

where “*a*” is a coefficient, *M* is the methylation level of each standard sample, Df is the Df value for each standard sample, and Df_max_ is the maximum Df value for the 100% methylated standard. The “nls” function in R 4.2.1 was used to implement the regression model and calculate the estimated value of “*a*” as in Qi et al. (2021). Typical standard curves for *NR1D1* and *CLDN22* are shown in Figure A5 in Appendix 1. Subsequently, the “predict” function in R 4.2.1 was used to generate a table of estimated methylation proportions for a range of Df values, that was then used to determine the methylation proportion of each sample.

To investigate the relationship between methylation proportion of the amplified regions within the promoter of *NR1D1* or within the TSS of *CLDN22* and *S. trachea* infection status, we fitted linear mixed models using lme4qtl (Ziyatdinov et al., 2018) with arcsine transformed methylation proportion as the response variable (Figure A6a,b in Appendix 1). Either infection status was included as a binary fixed factor in models, or fecal egg count (FEC) was included as a continuous covariate. Year and island were included as random factors to control for the effect of environment on methylation proportion, individual ID was included as a random factor to control for pseudoreplication because samples were run in duplicate, and the pedigree relatedness matrix was included as a random effect to control for kinship and population structure. Because rapid changes in DNA methylation levels are common in early development, particularly in altricial birds (Watson et al., 2019), we investigated whether methylation proportion at the amplified gene regions was related to age in days to evaluate the degree to which individual age could have impacted our analyses. Information on age in days was only available for the subset of individuals that were first recorded in the nest (*n* = 130 individuals for *NR1D1* and *n* = 124 individuals for *CLDN22*). Linear mixed models with arcsine transformed methylation proportion as the response variable, age in days as a continuous covariate, and infection status as a fixed factor were fitted for both *NR1D1* and *CLDN22*. Year, island, individual ID, and the pedigree relatedness matrix were again included as random factors in the models. Finally, if methylation proportion at NR1D1 or CLDN22 was related to infection status or FEC, we also investigated whether juvenile recruitment was related to methylation proportion at the amplified region of that gene. We used GlmmTMB (Brooks et al., 2017) to fit a generalized linear mixed model with recruitment as a binary response variable (0 if an individual did not recruit and 1 for recruits), infection status as a fixed factor, mean methylation proportion for sample duplicates as a continuous covariate, and an interaction between infection status and methylation proportion to explore whether plasticity in the immune response generated by methylation changes due to parasitism impacted recruitment. A relationship between juvenile recruitment probability and methylation proportion was expected for infected individuals, whereas no relationship was a priori expected for uninfected individuals. Year and island were included as random factors in the model.

## 3 RESULTS

**FIGURE 1:**
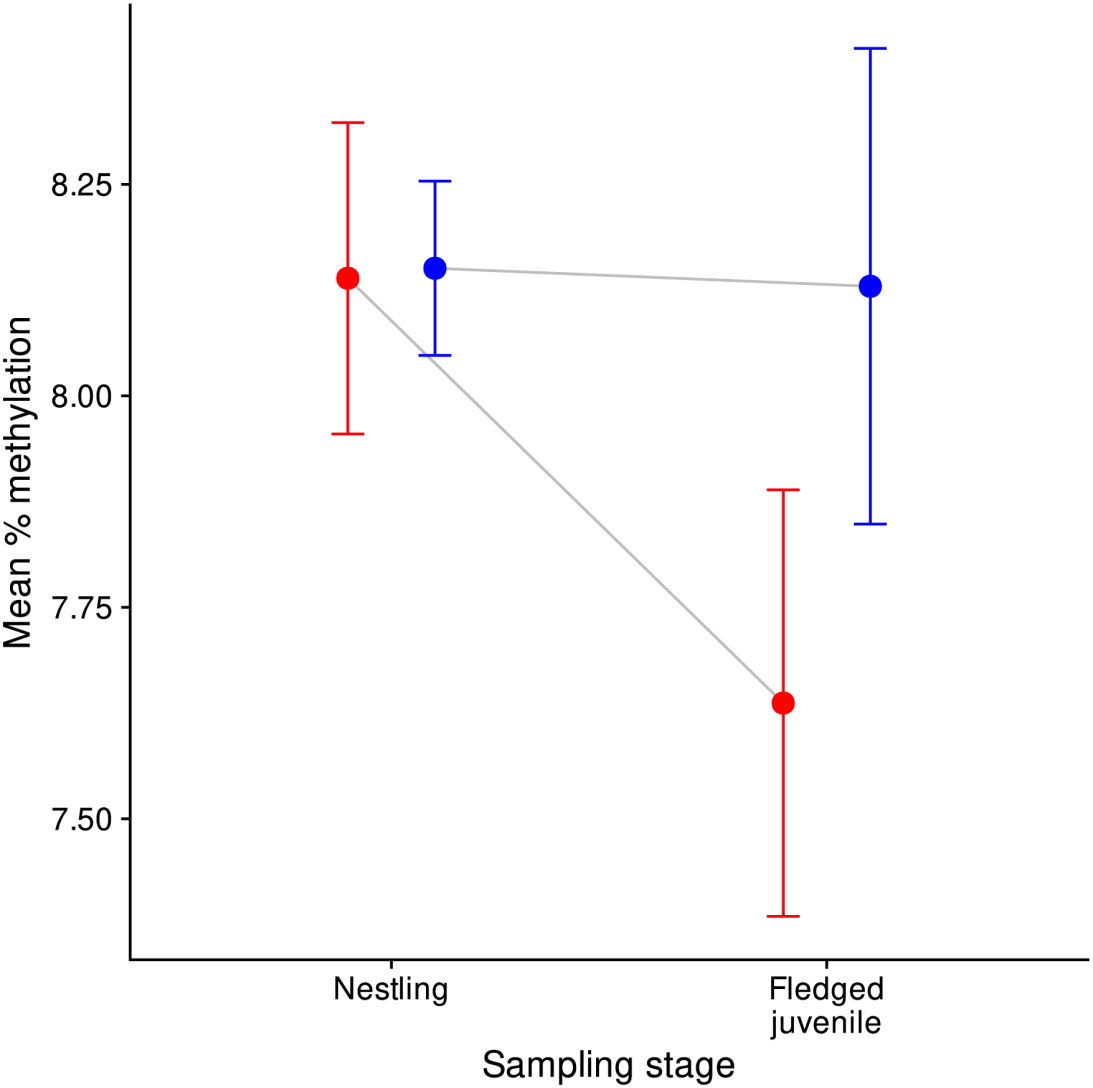
Mean genome‐wide methylation percentage in six cases and six control house sparrows that were sampled at both the nestling and fledged juvenile stage. Cases are shown in red and controls in blue. Each individual was sampled twice, once at the nestling stage prior to infection by *Syngamus trachea* and again at the fledged juvenile stage when case birds were infected by the parasite (*n* = 24 samples in total). Error bars represent standard error of the mean methylation across 337,524 shared 10× CpG sites between individuals. Mean methylation was slightly higher in nestlings and fledged juvenile controls than in fledged juvenile cases, and there was moderate evidence that individual mean methylation level decreased between the nestling and fledged juvenile stage in cases but not in controls (*p*‐values from paired *t*‐tests were .014 between nestling cases and fledged juvenile cases compared to .936 between nestling controls and fledged juvenile controls).

**FIGURE 2:**
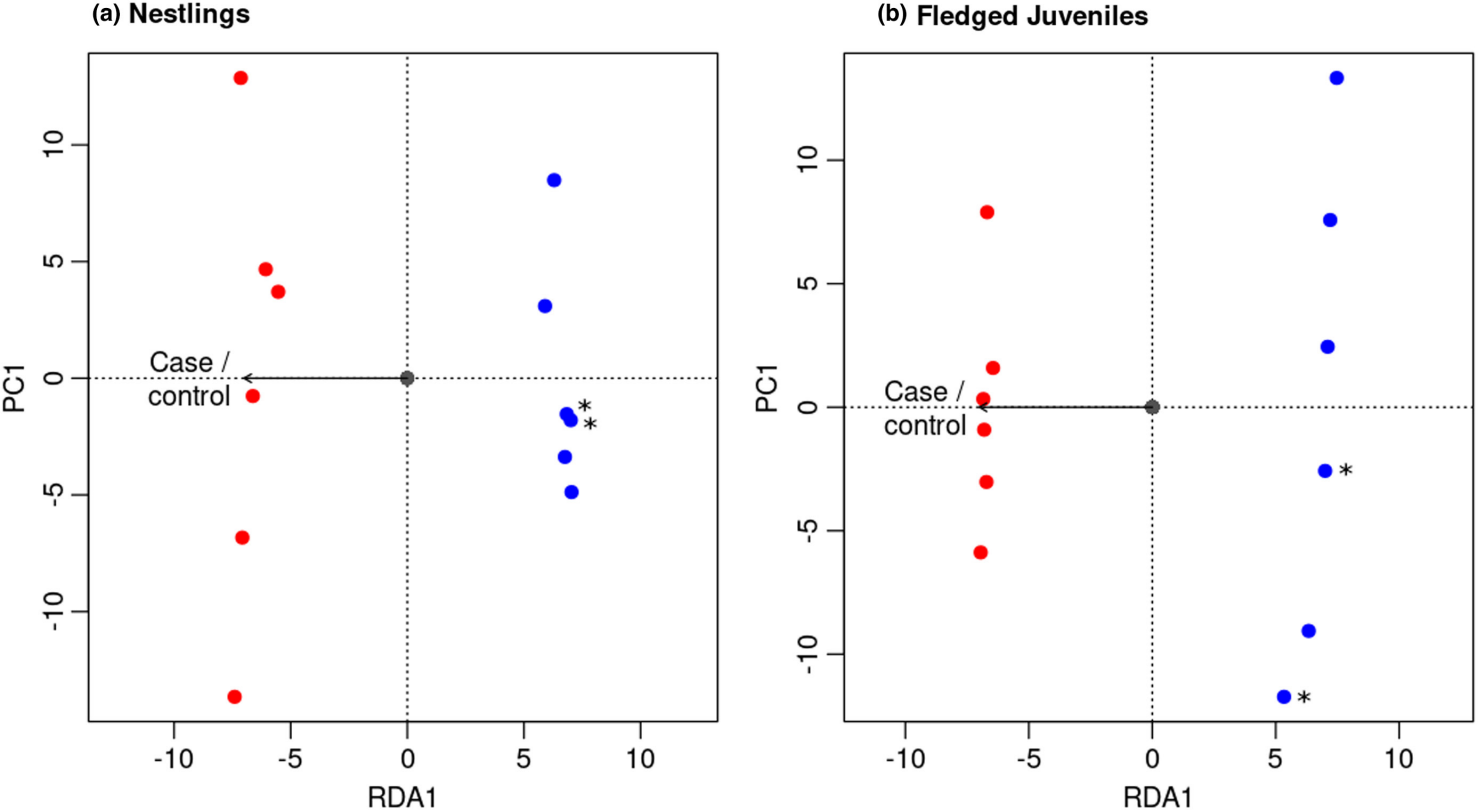
Redundancy analyses (RDA) of methylation profiles based on all CpG sites with shared 10× coverage in the juvenile dataset (337,524 sites). Each point represents one individual and each individual was sampled twice, once at the nestling stage and again at the fledged juvenile stage. RDA on DNA methylation profiles was performed separately on the nestling and fledged juvenile samples, with case–control group included as a fixed factor in both models. Cases are shown in red and controls in blue, and the full sibling pair is indicated by an asterisk at both stages. Case control group did not predict similarity of DNA methylation profiles between individuals at the nestling stage (*p* = .680, adjusted *R* ^2^ = −.001), nor at the fledged juvenile stage (*p* = .127, adjusted *R* ^2^ = .002), and the proportion of the variation in DNA methylation explained by case–control group at both the nestling and fledged juvenile stage was low.

**FIGURE A1:**
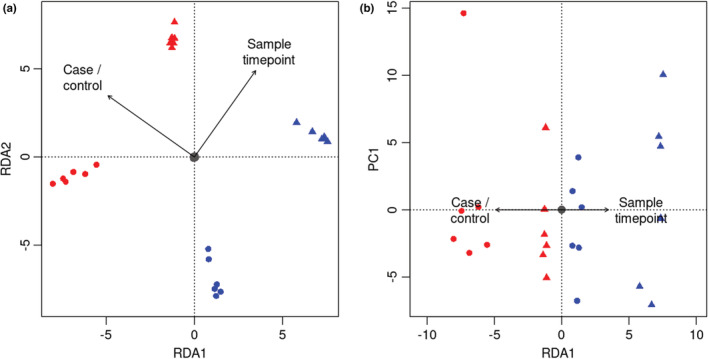
Redundancy analyses (RDA) of methylation profiles based on all CpG sites with shared 10× coverage in the juvenile dataset (337,524 sites). Each point represents one individual: Cases are shown in red and controls in blue, and each individual was sampled twice, once at the nestling stage (points) and again at the fledged juvenile stage (triangles). An RDA on methylation profiles was performed on all samples, with case–control group and stage (nestling or fledged juvenile) included as a fixed factors in the model. (a) Case–control group contributed more to variation in RDA1 than stage, whereas stage contributed more to variation in RDA2. There was evidence that RDA1 was related to genome‐wide methylation levels (RDA1 *p* = .015, RDA2 *p* = .562). However, due to pseudoreplication (the same individuals were sampled at both the nestling and fledged juvenile timepoints) the significance of this result should be interpreted with caution, and the proportion of the variation in methylation levels explained by case–control group and stage was low (adjusted *R* ^2^ = .005). (b) the first constrained axis (RDA1) plotted against the first unconstrained axis (PC1). Here, samples cluster by case–control group as well as by stage, although PC1 contributed more to the variance in genome‐wide methylation levels than RDA1.

The mean mapping efficiency across all RRBS libraries was ~80% (Table A1 in Appendix 1), and, after filtering, 337,524 CpG sites with shared 10× coverage were available for downstream analyses in the juvenile dataset (Table A2 in Appendix 1). Mean methylation level was slightly higher in nestlings and fledged juvenile controls than in fledged juvenile cases, although this difference was not significant (Figure 1, *p*‐values from unpaired *t*‐tests were .957 between nestling cases and controls, and .222 between fledged juvenile cases and controls), and the absolute methylation difference between groups was small. There was moderate evidence of a shift in methylation patterns within the same individuals following infection (Figure 1, *p*‐values from paired *t*‐tests were .014 between nestling cases and fledged juvenile cases compared to .936 between nestling controls and fledged juvenile controls). However, redundancy analyses in the juvenile dataset (Figure 2) suggested that infection status explained little of the variance in genome‐wide methylation levels (*p* = .680 and adjusted *R* ^2^ = −.001 at the nestling stage, *p* = .127 and adjusted *R* ^2^ = .002 at the fledged juvenile stage), which is unsurprising because only a small proportion of genome‐wide CpG sites are expected to be in the TSS, promoter, or first intron of genes related to parasite defense. When RDA was performed on nestling and fledged juvenile samples together, infection status was a better indicator of similarity of methylation profiles than stage (Figure A1 in Appendix 1) but still explained very little of the variance in genome‐wide methylation levels (RDA1 *p* = .015, adjusted *R* ^2^ = .005). Of the CpG sites that showed at least 15% methylation difference between cases and controls in fledged juveniles, 5455 of these sites had 10× coverage in the adult dataset and could be used to determine whether the infection status of adult house sparrows was related to methylation profile at these sites (Table A2 in Appendix 1). There was, however, little evidence that adult cases had lower mean methylation levels than adult controls (unpaired *t*‐test *p*‐value = .558; Figure A3 in Appendix 1) and RDA (Figure A4 in Appendix 1) showed that infection status did not contribute to the variance in methylation profile in adult house sparrows at these sites (*p* = .943, adjusted *R* ^2^ = −.006).

### 3.1 The effect of parasite infection on methylation patterns

**FIGURE 3:**
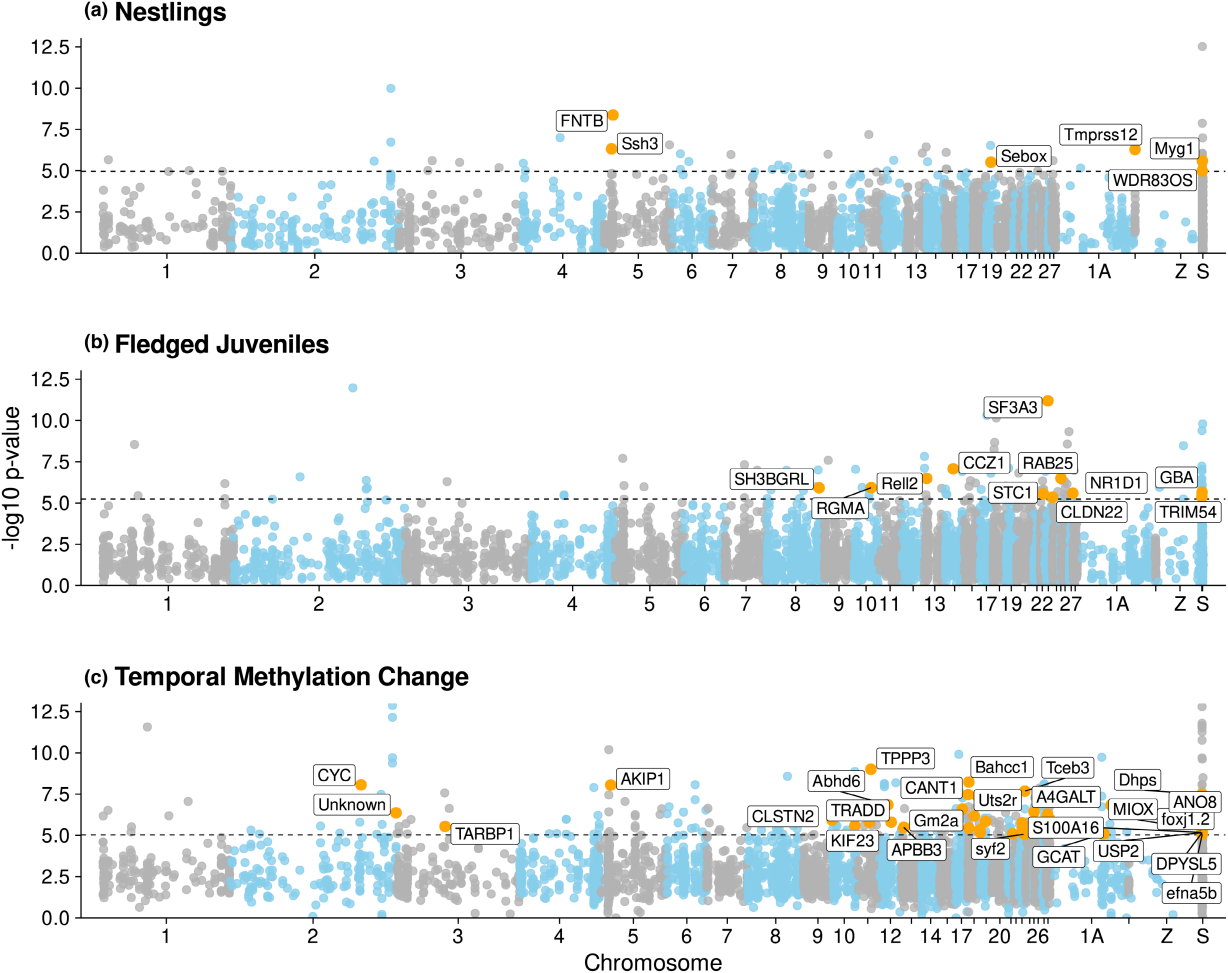
Differential methylation analysis results. Only CpG sites that showed at least 15% methylation difference between cases (*n* = 6) and controls (*n* = 6) were analyzed, and for all models, Bonferroni correction with a FWER of 0.05 was used to identify differentially methylated cytosines (DMCs). “LGE22” is a linkage group corresponding to part of an unknown chromosome, “Z” is the sex chromosome, and “S” represents all scaffolds for which chromosome location is unknown. For (a) nestlings 56 of 4530 analyzed CpG sites were differentially methylated between cases and controls. For (b) fledged juveniles, 120 of 8834 analyzed CpG sites were differentially methylated between cases and controls. (c) Difference in temporal change in methylation level between cases and controls was also observed for 289 of 5410 analyzed CpG sites. Differentially methylated cytosines (DMCs) in the TSS, promoter, or first intron are highlighted in orange and labeled with corresponding genes.

At the nestling stage, 56 of 4530 analyzed CpG sites showed evidence of differential methylation between cases and controls after Bonferroni correction (Figure 3a), and 120 of 8834 analyzed sites were differentially methylated between cases and controls at the fledged juvenile stage (Figure 3b). We also found support for differences in temporal change in methylation levels between cases and controls, indicated by evidence of an interaction between infection status and stage for 289 of 5410 analyzed CpG sites (Figure 3c). See Table 3 for an overview of the number of differentially methylated sites detected in each differential methylation analysis, as well as mean methylation percentage at these sites in cases and controls.

**TABLE 3:** Number of CpG sites assigned to different models in differential methylation analyses in lme4qtl, and mean methylation percentage ± SE for cases and controls separately.

	Number of sites	Methylation % cases	Methylation % controls
Nestlings	56	39.080 ± 3.099	40.333 ± 3.512
Fledged juveniles	120	34.804 ± 2.113	42.590 ± 2.238
Temporal methylation change	289	Δ −9.459 ± 0.796	Δ 6.511 ± 0.839

### 3.2 Genomic locations and gene annotation

**FIGURE 4:**
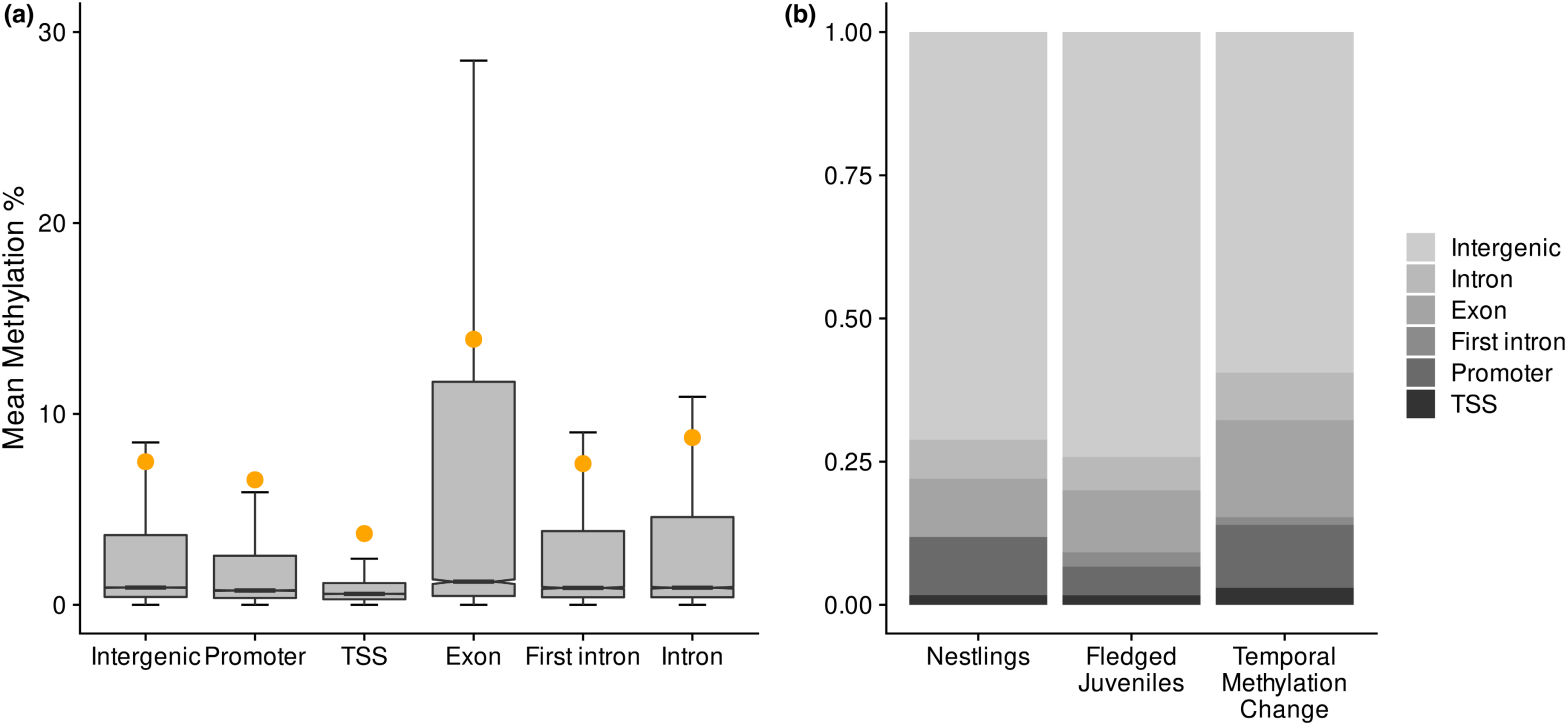
(a) Boxplot of methylation levels at different genomic features, based on annotating 337,524 shared 10× CpG sites against the *Passer domesticus* genome v 1.0. There was very strong evidence that all distributions were different from each other (*p* < 2^−16^) based on results of pairwise Wilcoxon rank sum tests, except for the comparison between introns and intergenic regions (*p* = .240; see Table A3 in Appendix 1), first introns and intergenic regions (*p* = .290), and first introns compared to other introns (*p* = .037). The orange point above each box indicates the mean methylation level, and the horizontal black line represents median methylation level. (b) Genomic locations of differentially methylated CpG sites from differential methylation analyses. For all differential methylation analyses, the percentage of CpG sites assigned to each genomic feature (i.e. intergenic, promoter, transcription start site (TSS), exon, first intron, or intron) was similar between all analyzed versus differentially methylated sites.

CpG sites shared between all individuals at the nestling and fledged juvenile stage were assigned to the promoter, TSS, exon, first intron, or other introns of genes or were categorized as intergenic. Methylation levels were highest in exons, introns, and intergenic regions, and lowest in the TSS and promoter (Figure 4a). There was very strong evidence that methylation levels differed between all genomic features, except for introns and intergenic regions (pairwise Wilcoxon rank sum tests, see Table A3 in Appendix 1). Methylation differences at CpG sites in the TSS or promoter have been shown to influence gene expression in passerines (Laine et al., 2016), and a consistent negative relationship between DNA methylation levels in the first intron of genes and gene expression has been demonstrated in several species (Anastasiadi et al., 2018). As such, any DMCs assigned to these genomic features are of particular interest. Of the DMCs that were differentially methylated between cases and controls at the nestling stage, 1.7% were located in the TSS, 12.8% were located in the promoter, and 0.0% were located within the first intron of genes (Figure 4b). Of the DMCs that were differentially methylated between cases and controls at the fledged juvenile stage, 1.6% were located in the TSS, 5.0% were in promoters, and 2.5% were within the first intron (Figure 4b). Furthermore, 3.0% of the DMCs where temporal change in methylation level was different between cases and controls were located in the TSS, 11.0% were in promoters, and 1.4% were within the first intron (Figure 4b). Proportion of CpGs assigned to each genomic location was similar between all analyzed CpGs and DMCs. A majority of the genes that had DMCs in their TSS, promoter, or first intron have known immunomodulatory or mucus membrane integrity functions, see Table A4 in Appendix 1 for details.

### 3.3 Functional analysis

Only genes with DMCs in the TSS, promoter, or first intron were included in functional analysis. No functional terms were enriched in functional analysis on the candidate genes identified for nestlings. The GO Biological Process term “epithelial cell morphogenesis” (GO:0003382) was enriched in functional analysis on the genes identified for fledged juveniles when using both human and chicken gene ontologies. Three functional groups were enriched in functional analysis on the candidate genes identified in the temporal differential methylation analysis (see Table A5 in Appendix 1 for a full list of enriched terms and their associated genes). These included Reactome relevant pathways relating to TNFR1, NF‐κB, and TNF signaling (RhasA:5357786has‐HSA:5357956, R‐HSA:75893, R‐HSA:5357905), as well as the GO Biological Process terms “eye photoreceptor cell differentiation” (GO:0001754), “positive regulation of axon extension” (GO:0045773) and “eye photoreceptor cell development” (GO:0042462). The same GO terms were enriched when using chicken gene ontologies, however, Reactome Pathways was not available for chicken.

### 3.4 Candidate gene verification using MS‐HRM

Using MS‐HRM, we were able to investigate the relationship between methylation proportion at two of our candidate genes and parasitism by *S. trachea* in a larger dataset that included samples from 322 fledged juvenile house sparrows (Tables 2 and 4). There was very strong evidence that methylation proportion at the amplified region within the promoter of *NR1D1* was higher in individuals infected by *S. trachea* than in uninfected individuals (*Δ*_infected‐uninfected_ = 0.094, *p* = .001), but there was no evidence that methylation proportion at *NR1D1* was related to FEC (*β* = 0.000, *p* = .802). There was no evidence that methylation proportion at the amplified region within the TSS of *CLDN22* was related to infection status (*Δ*_infected‐uninfected_ = 0.001, *p* = .949) or FEC (*β* = 0.000, *p* = .890). However, in the subset of individuals with information on age in days at the time of sampling (Table A7 and Figure A6c,d in Appendix 1), there was moderate evidence that methylation proportion at *CLDN22* was positively related to age in days (*β* = 0.0004, *p* = .023) although the effect size was small, whereas there was no evidence that methylation proportion at *NR1D1* was related to age in days (*β* = 0.001, *p* = .165).

**TABLE 4:** Results of linear mixed‐effects models using the MS‐HRM dataset to validate the relationship between methylation proportion at the amplified regions within the promoter of NR1D1, or within the TSS of *CLDN22*, and parasitism by *Syngamus trachea*.

Gene	Infection status	FEC	Relmat.	ID	Year	Island	Residual
NR1D1	0.094 (.001)	‐	0.016 (0.126)	0.035 (0.187)	0.006 (0.080)	0.000 (0.000)	0.012 (0.111)
	‐	0.000 (.802)	0.020 (0.143)	0.033 (0.181)	0.005 (0.073)	0.000 (0.000)	0.012 (0.111)
CLDN22	0.001 (.949)	‐	0.002 (0.046)	0.001 (0.034)	0.000 (0.012)	0.000 (0.011)	0.000 (0.013)
	‐	0.000 (.890)	0.002 (0.046)	0.001 (0.034)	0.000 (0.012)	0.000 (0.011)	0.000 (0.013)

*Note*: Effect size (above) and *p*‐value (below in parenthesis) are given for the main effects of infection status or fecal egg count (FEC). Variance (above) and standard deviation (below in parenthesis) are given for the random factors pedigree relatedness (Relmat), individual ID, year, island, and the residual variance.

Because methylation proportion at *NR1D1* was positively related to *S. trachea* infection status, we investigated whether juvenile recruitment probability was related to methylation proportion at *NR1D1* or to infection status (Table 5). There was little evidence of an interaction effect of methylation proportion and infection status on recruitment probability (*β* = −0.274, *p* = .869), and there was no evidence of a main effect of methylation proportion (*β* = 2.341, *p* = .120) or infection status (*Δ*_infected‐uninfected_ = −0.151, *p* = .670) on recruitment probability. Because a large noise signal for the effect of methylation on recruitment in uninfected individuals could mask any relationship between recruitment probability and methylation proportion in infected individuals, we subsequently performed separate analyses on infected and uninfected individuals (Table 5). In the infected subset of individuals (*n* = 198) there was strong evidence that recruitment probability was positively related to methylation proportion (*β* = 2.097, *p* = .007), whereas in uninfected individuals (*n* = 113) there was no evidence that recruitment probability was related to methylation proportion (*β* = 2.374, *p* = .103).

**TABLE 5:** Results of generalized linear mixed‐effects models using the MS‐HRM dataset to investigate the relationship between recruitment probability and individual mean methylation proportion at the amplified region within the promoter of *NR1D1*.

Dataset	Methylation proportion	Infection status	Meth. × infection status	Year	Island
Combined	2.341 (.120)	−0.151 (.670)	−0.274 (.869)	0.145 (0.381)	0.067 (0.259)
Infected	2.097 (.007)	‐	‐	0.253 (0.503)	0.073 (0.271)
Uninfected	2.374 (.103)	‐	‐	0.000 (0.000)	0.010 (0.099)

*Note*: We first ran the model on the entire dataset (*n* = 322 individuals) and included an interaction effect of methylation proportion and infection status to investigate whether plasticity in the immune response generated by methylation changes due to parasitism impacted recruitment. Subsequently, we ran the model separately on infected and uninfected individuals. Effect size (above) and *p*‐value (below in parenthesis) are given for the main effects of methylation proportion and infection status on recruitment probability, as well as for the interaction effect of methylation proportion and infection status on recruitment probability. Variance (above) and standard deviation (below in parenthesis) are given for the random factor year and island.

## 4 DISCUSSION

Although there is increasing evidence from controlled experiments that epigenetic modifications such as DNA methylation may mediate phenotypic plasticity in the response to pathogen infection, the contribution of epigenetic mechanisms to parasite resistance remains largely unexplored in natural populations. Here, we expand on current knowledge by indicating a role for DNA methylation in mounting the immune response in house sparrows infected by the parasitic nematode, *S. trachea*. We used our RRBS dataset to investigate whether methylation differences between house sparrow nestlings was associated with the probability of later infection by the parasite, and if infection at the fledged juvenile stage affected genome‐wide DNA methylation patterns. We found that genome‐wide DNA methylation profiles were similar between cases and controls at the nestling stage, while DNA methylation levels were slightly lower in infected house sparrows. Furthermore, multiple immune pathways were identified in differential methylation and functional analyses, which suggests that DNA methylation may play a role in the immune response to parasite infection. Subsequently, we used MS‐HRM analyses and a larger sample dataset to validate the relationship between methylation proportion and *S. trachea* infection status for two candidate genes, *NR1D1* and *CLDN22*, that were identified in differential methylation analysis on the fledged juvenile RRBS dataset. We found that methylation proportion at *NR1D1*, but not at *CLDN22* remained related to infection status, and that recruitment probability of fledged juveniles infected by *S. trachea* was positively related to methylation levels at *NR1D1*. This underscores the importance of performing follow‐up studies on putative candidate genes, and ideally the need for functional studies on methylation levels at candidate genes (Gudmunds et al., 2022; Husby, 2020, 2022).

### 4.1 Genome‐wide DNA methylation patterns

**FIGURE A2:**
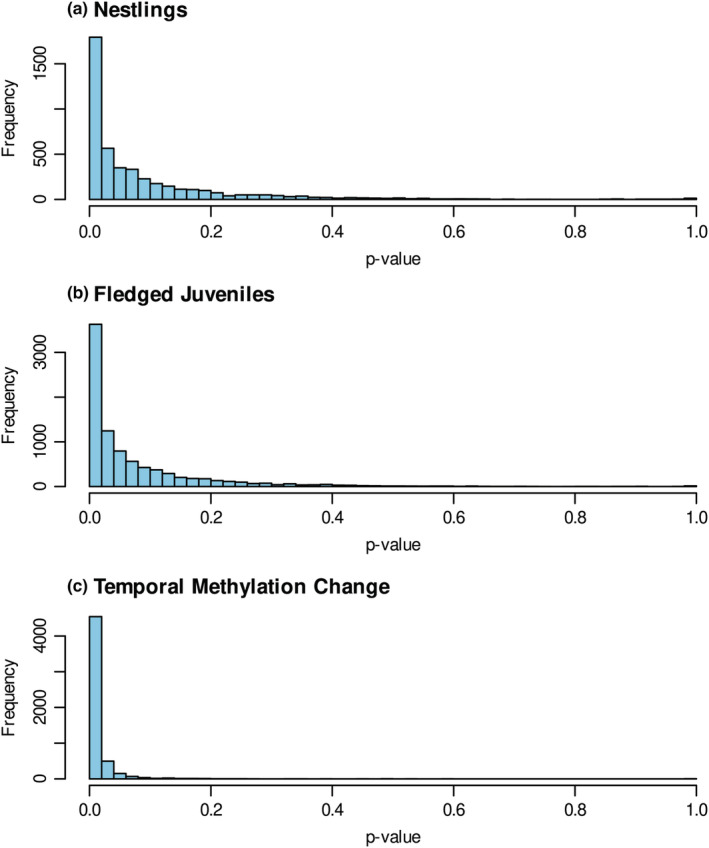
*p*‐Value distributions from differential methylation analyses in lme4qtl. For differential methylation analysis we selected sites with at least 15% difference between cases and controls for the nestling and fledged juvenile timepoints, or with at least 15% Δ methylation difference between cases and controls in the temporal analysis.

**FIGURE A3:**
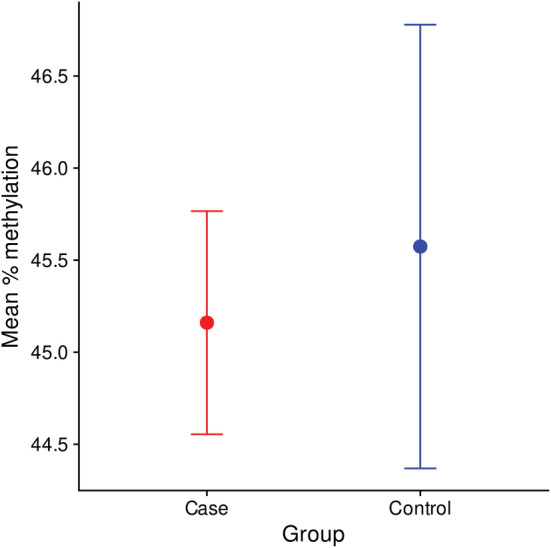
Mean methylation percentage in adult samples at CpG sites that showed at least 15% methylation difference between cases and controls in fledged juveniles. Cases are shown in red and controls in blue. Error bars represent standard error of the mean methylation between individuals across 5455 sites that had shared 10× coverage between all adult samples. There was little evidence that mean methylation across these sites was lower in adult cases than adult controls (*p*‐value from an unpaired *t*‐test = .558).

**FIGURE A4:**
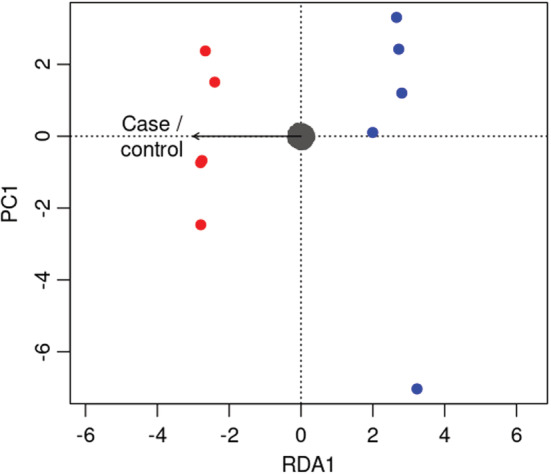
Redundancy analyses (RDA) of methylation profiles based on the 5444 CpG sites that showed at least 15% methylation difference between cases and controls in fledged juveniles and that had at least 10× coverage in the adult dataset. Each colored point represents one adult individual, infected individuals are shown in red and controls in blue. Infection status did not predict similarity of methylation profiles at these sites in adult birds (*p* = .943) and explained little of the variation in methylation levels between individuals (adjusted *R* ^2^ = −.006).

**FIGURE A5:**
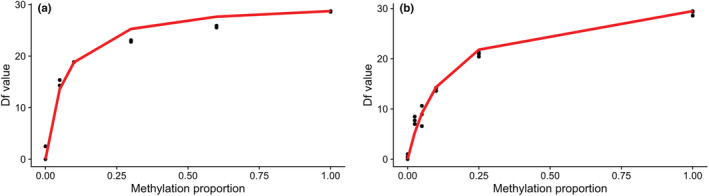
Typical standard curves for (a) *NR1D1* (nuclear receptor subfamily 1 group D member 1) and (b) CLDN22 (Claudin 22). Df is the maximum absolute value of the relative fluorescence signal differences for each sample, based on the raw fluorescence data from methylation‐sensitive high‐resolution melting (MS‐HRM).

DNA methylation levels were highest in exons followed by intergenic regions and introns, lower in promoters, and lowest in the TSS of genes (Figure 4a, Table A3 in Appendix 1). This is similar to methylation patterns found in previous genome‐wide DNA methylation studies in passerines (Laine et al., 2016; Viitaniemi et al., 2019). At the nestling stage, mean methylation levels were similar between cases (individuals that were later infected by *S. trachea* at the fledged juvenile stage) and controls (individuals that were not infected by *S. trachea* during their first year of life). However, some DNA methylation differences were observed at the fledged juvenile stage where infected birds had slightly lower methylation levels compared to uninfected controls, and mean methylation decreased to a greater extent in cases compared to controls between the two stages (Figure 1). This suggests that infection of house sparrows by *S. trachea* could result in genome‐wide DNA methylation changes. Nonetheless, the results of redundancy analyses suggested that infection status explained little of the variance in genome‐wide methylation levels. Similar findings were demonstrated in a study that used experimental manipulation of ectoparasite levels in free‐living mockingbirds and captive zebra finches (McNew et al., 2021), where no differences in genome‐wide methylation levels but clear CpG site‐specific differences between treatments were found. An earlier study on epigenetic response of wild grouse to parasitic nematode infection (Wenzel & Piertney, 2014) also detected no difference in genome‐wide methylation levels between infected and control birds, however, the AFLP method that was used in the above study is less sensitive than RRBS. The slight divergence in the methylation profiles of infected birds that was demonstrated at the fledged juvenile stage in the present study is in contrast to the findings in Sagonas et al. (2020), where intense parasite pressure triggered a pronounced and coordinated epigenetic response to parasitic nematode infection in the stickleback. Due to the environmentally responsive nature of DNA methylation (Angers et al., 2010; Hu & Barrett, 2017), it is important to control for environmental variation in DNA methylation analyses. Thus, the environmental variables sampling location and year were kept constant in our juvenile RRBS dataset to increase power to detect any epigenetic differences between case and control birds (Table 1). Nonetheless, it is possible that differences in micro‐environment between individuals could have influenced DNA methylation levels and impacted our results. In adult birds, infected and uninfected individuals showed no evidence of epigenetic differentiation at CpG sites that had at least 15% methylation difference between cases and control in fledged juveniles (Figures A2, A3, A4 in Appendix 1). All uninfected adults were feces sampled at least once as juveniles and had a FEC of zero on all feces sampling occasions. Thus, the lack of methylation difference between infected and uninfected adults is unlikely to be a result of long‐lasting DNA methylation changes following any early‐life infection. Re‐infection by *S. trachea* in our study system is rare as only approximately 1% of birds are re‐infected (Holand et al., 2013), and an adaptive immune response to infection by the parasite has previously been implicated (Lundregan et al., 2020). Therefore, the epigenetic response to infection mounted by adult birds may differ from that of juveniles, and epigenetic differences due to factors such as developmental stage (Watson et al., 2019) or reproductive status at different points in the breeding season (Lindner, Laine et al., 2021; Viitaniemi et al., 2019) could also contribute to genome‐wide differences in methylation in adult house sparrows.

### 4.2 Genomic locations of differentially methylated cytosines

We observed differential methylation in the TSS, promoter, or first intron of immune genes at both the nestling and fledged juvenile stage and also identified several genes where temporal change in methylation level differed between cases and controls. The majority of CpG sites that were differentially methylated according to infection status were annotated to introns, exons, and intergenic regions rather than the TSS, promoter, or first intron. This result is similar to those from previous studies that performed RRBS using avian DNA from red blood cells (Pértille et al., 2017; Viitaniemi et al., 2019). In the current study, 6.6%–14.5% of DMCs were mapped to the TSS or promoter (Figure 4b), which is similar to the proportion of DMCs mapped to these genomic features in previous studies on DNA methylation during parasite infection (McNew et al., 2021; Sagonas et al., 2020). The candidate genes that were identified in the present study had functions relating to both innate and adaptive immune response, as well as mucus membrane integrity and physical degradation of parasites (Table A4 in Appendix 1), in agreement with results in Lundregan et al. (2020). In nestlings, genes relating to T‐cell homeostasis (*FNTB*, Du et al., 2020) as well as initial immune responses including autophagy (Ssh3, Pinto et al., 2021), release of P‐selectin (*Rab15*, Nightingale & Cutler, 2013; Prashar et al., 2017), and serine protease (*Tmprss12*, Molehin et al., 2012), and production of reactive oxygen species (ROS: *Myg1*, Grover et al., 2019; Isaksson et al., 2013) were identified. Therefore, although no genome‐wide differences in methylation patterns between cases and controls were detected at the nestling stage prior to infection by *S. trachea*, DNA methylation differences at specific genes involved in regulation of immune homeostasis and the initial immune response to pathogens may be a factor in increased susceptibility of some house sparrows to subsequent parasite infection.

Conversely, several genes linked to activation of the innate and adaptive immune responses were identified at the fledged juvenile stage (Table A4 in Appendix 1), when cases were infected by *S. trachea* while controls remained uninfected. Infected birds had lower methylation levels at *TRIM54*, which is involved in TLR4 signaling (Jefferies et al., 2011), as well as *SF3A3*, a negative regulator of Toll‐like receptor (TLR) signaling (De Arras & Alper, 2013). Infected birds also had higher methylation levels at *NR1D1*. *NR1D1* encodes the protein REV‐ERBα that is a negative regulator of immune genes including, *TLR4*, *IL‐6*, *IL‐1β*, and *Nlrp3*. REV‐ERBα is also involved in NF‐κB signaling and transcription of inflammation‐related genes (Liu et al., 2020; Wang et al., 2020). Furthermore, infected birds had methylation changes at genes controlling CD4+ cell activation (*RGMA*, Fujita & Yamashita, 2017), and B‐cell and T‐cell production, activation, and maturation (*Rell2*, Sica et al., 2001; *GBA*, Liu et al., 2012). Taken together, these results suggest that there might be both an innate and adaptive immune component to defense against *S. trachea* that may be modulated by DNA methylation at specific immune genes. Two genes related to epithelial integrity were also differentially methylated between infected birds and controls. Methylation levels at *CLDN22* that regulates epithelial permeability in the lung and intestine were higher in infected birds. Viruses and bacteria (Soini, 2011) as well as helminth parasites (Su et al., 2011) have been shown to increase epithelial permeability by downregulating expression of Claudins. Methylation levels at STC1 were lower in cases than controls, and upregulation of this gene has been related to wound closure in lung epithelium (Ito et al., 2014).

Genes for which the temporal change in methylation level was different between cases and controls had a broad range of functions (see Table A4 in Appendix 1 for a complete overview). Case and control birds diverged epigenetically at genes relating to apoptosis (*Nucleoside diphosphate kinase*, *efna5b*, *CYC*, see Eleftheriadis et al., 2016; Park et al., 2013; Schlattner et al., 2009), innate mucosal immunity (*CANT1*, Arase et al., 2009; Calì et al., 2012), and generation of reactive oxygen species (ROS: *Gm2a*, *MIOX*, see Borges et al., 2017; Gonzalez‐Uarquin et al., 2021; Inohara & Nuñez, 2002). Several differentially methylated genes were involved in pattern recognition receptor (PRR) activation and signaling (*med16*, *A4GALT*, and *Hmg20b*, see Kim et al., 2004; Kondo et al., 2013; Zhang & Cao, 2019), a process whereby PRRs recognize pathogen‐derived molecules to initiate the immune response. Another detected gene, *CYC*, is a mitochondrial damage‐associated molecular pattern (DAMP) that is recognized by PRRs upon cell injury (Eleftheriadis et al., 2016). PRR signaling commonly activates the canonical NF‐κB pathway and *AKIP1* that showed evidence of temporal differential methylation regulates the rate of NF‐κB nuclear translocation (King et al., 2011). *Rab11A*, a regulator of TLR4 transport (Husebye et al., 2010), was also identified. Temporal differential methylation analyses also identified genes related to adaptive immune processes including CD4+, T‐cell, and B‐cell expression and maturation (*VPREB3*, *TPPP3*, see Rosnet et al., 2004; Yang et al., 2020). These findings are in agreement with Perrigoue et al. (2008) who describe the innate immune response to helminth infection that leads to initiation and maintenance of adaptive immune responses. Interestingly, several temporally differentially methylated genes that contribute to histone methylation were also identified (*LSM10*, *Hmg20b*, *PRDM2*, *Bahcc1*, see Fan et al., 2020; Khurana et al., 2014; Kooistra & Helin, 2012; Marzluff et al., 2008).

### 4.3 Functional analysis

Results of GO functional analysis supported those from differential methylation analyses. In functional analysis of the candidate genes identified for fledged juveniles, the term “epithelial cell morphogenesis” was enriched. Many of the mesenchymal signaling pathways involved in epithelial morphogenesis may also be involved in epithelial repair after injury (Fang, 2000), and STC1 that was associated with this functional group has been related to wound closure in lung epithelium (Ito et al., 2014). In functional analysis of the candidate genes from the temporal differential methylation analysis, the functional group with the lowest *p*‐value included terms related to TNFR1, NF‐κB, and TNF signaling. These signaling pathways are interconnected and regulate immune function by inducing expression of pro‐inflammatory cytokines and governing survival, activation, and differentiation of immune cells (Liu et al., 2017; Zhang & Cao, 2019). Thus, the enrichment of these functional terms suggests that processes relating to epithelial wound repair may be important in minimizing any mechanical damage of tracheal epithelium caused by *S. trachea* and that DNA methylation changes at immune genes may play a central role in mounting an immune response to parasite challenge.

### 4.4 Candidate gene verification using MS‐HRM

**FIGURE 5:**
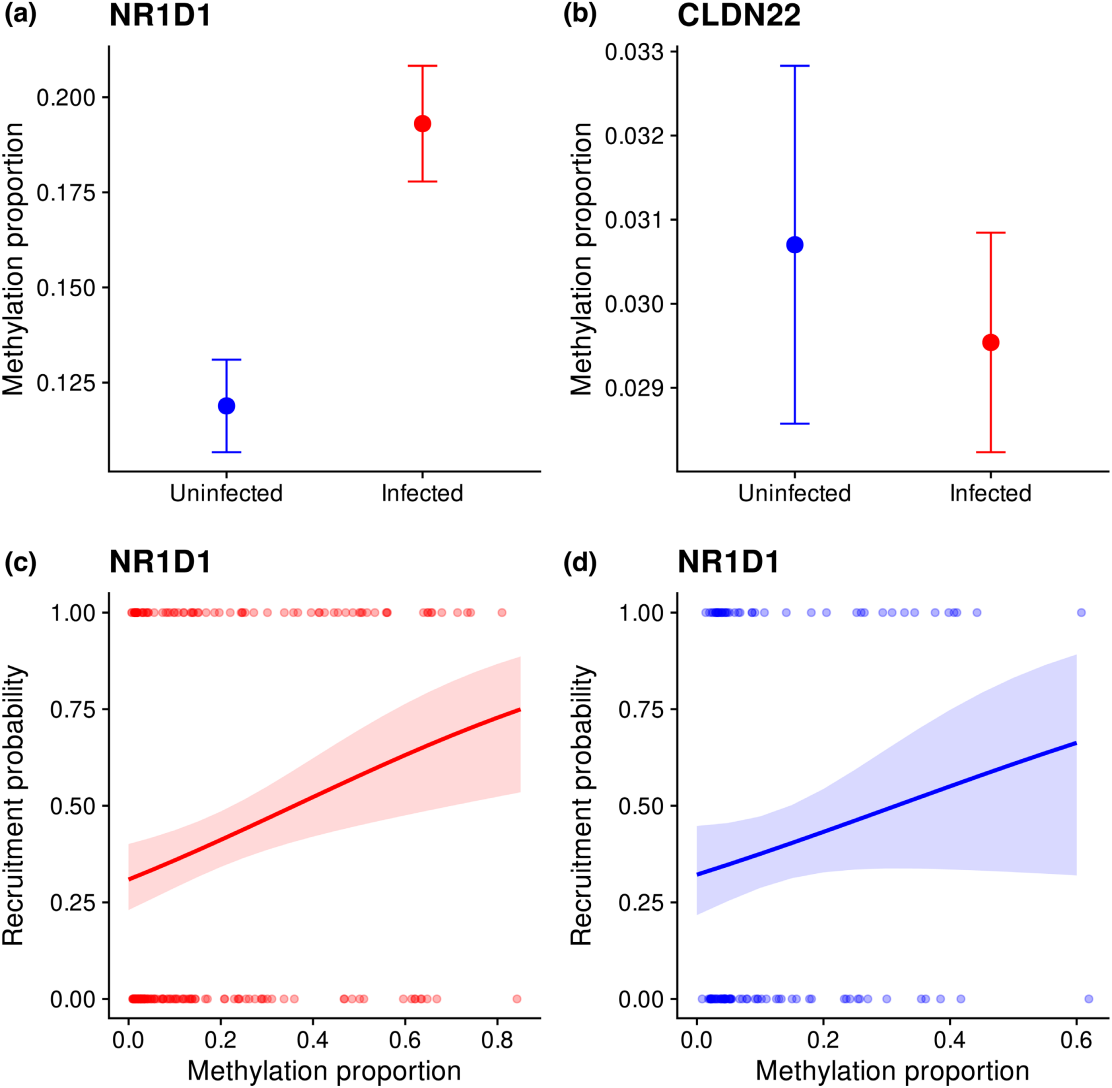
Results of MS‐HRM analyses. (a) Mean methylation proportion ± SE according to infection status (uninfected = blue, infected = red) at the amplified region within the promoter of *NR1D1*. (b) Mean methylation proportion ± SE according to infection status (uninfected = blue, infected = red) at the amplified region within the TSS of *CLDN22*. (c) There was strong evidence that juvenile recruitment probability was positively related to methylation proportion at *NR1D1* in infected individuals (*β* = 2.097, *p* = .007). (d) There was little evidence that juvenile recruitment probability was related to methylation proportion at *NR1D1* in uninfected individuals (*β* = 2.374, *p* = .103).

Subsequently, we investigated the relationship between methylation proportion within the TSS or promoter region of two of our candidate genes (*NR1D1* and *CLDN22*) and parasitism by *S. trachea* using MS‐HRM analyses and a dataset that included samples from 322 fledged juvenile house sparrows (Table 2). We found that methylation proportion of the amplified region within the promoter of *NR1D1* was positively related to infection status (Table 4, Figure 5a), but unrelated to FEC, and was 64% greater in infected individuals (methylation proportion was 0.12 in uninfected individuals compared to 0.19 in infected individuals). The protein encoded by *NR1D1* is a negative regulator of immune genes including, *TLR4*, *IL‐6*, *IL‐1β*, and *Nlrp3* and is involved in NF‐κB signaling and transcription of inflammation‐related genes (Wang et al., 2020). Thus, higher methylation levels at *NR1D1* in infected individuals may have occurred as part of the immune response to *S. trachea* to enable transcription of key immune genes. Methylation proportion at *NR1D1* was unrelated to age in days in the subset of individuals for which we had age information (Table A7 in Appendix 1), so the observed relationship between methylation proportion at *NR1D1* and infection status is unlikely to be an artifact of the rapid change in methylation levels that is commonly observed during early development (Watson et al., 2019). Furthermore, we found strong evidence that juvenile recruitment probability was positively related to methylation proportion at *NR1D1* in infected individuals, but not in uninfected individuals (Table 5, Figure 5c,d), which is as expected because immune activation is costly (Graham et al., 2005; Råberg et al., 2009) and resource allocation to immune defense commonly trades off with other physiological processes such as growth (Tompkins et al., 2011). Infection status did not impact juvenile recruitment probability (Table 5), which, although unexpected, is in agreement with previous work in our study system that found no relationship between house sparrow survival probability and *S. trachea* infection status, but instead found that survival probability was negatively related to infection severity (Holand et al., 2015). The results of the present study suggest that DNA methylation differences at relatively few immune genes could influence recruitment probability of juvenile house sparrows infected by *S. trachea* and that DNA methylation changes may alter the fitness costs of parasitism in our study system.

**FIGURE A6:**
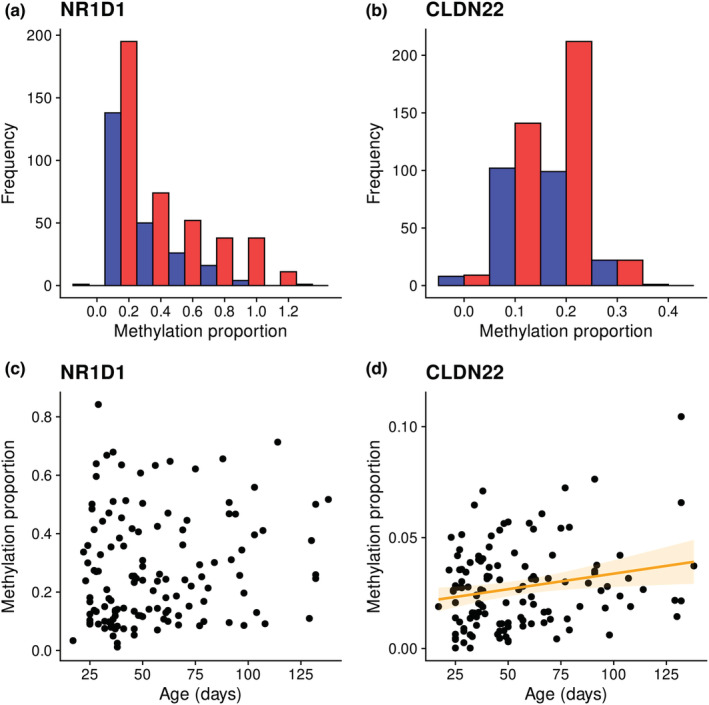
Frequency distribution histograms of the arcsine transformed methylation proportion data for (a) NR1D1, and (b) CLDN22. Distributions for infected individuals are shown in red and for uninfected individuals are shown in blue. (c) Results of the linear mixed model with arcsine transformed methylation proportion at NR1D1 as the response variable found no evidence of a relationship between methylation proportion and individual age in days (*β* = 0.001, *p* = .165). (d) Results of the linear mixed model with arcsine transformed methylation proportion at CLDN22 as the response variable found moderate evidence of a relationship between methylation proportion and individual age in days (*β* = 0.0004, *p* = .023).

Conversely, we found no evidence that methylation proportion of the amplified region within the TSS of *CLDN22* was related to either *S. trachea* infection status or FEC. Although we found moderate evidence that methylation proportion at *CLDN22* was positively related to age in days (Table A7 and Figure A6d in Appendix 1), the effect size was small, which suggests that age differences between juvenile house sparrows were unlikely to have impacted our results. Thus, the DMC within the TSS of *CLDN22* that was identified in our differential methylation analysis on the RRBS dataset is likely to be a false‐positive, despite previous studies that found that diverse pathogens (Soini, 2011) and helminth parasites (Su et al., 2011) can increase epithelial permeability by downregulating expression of Claudins. Due to constraints relating to MS‐HRM primer design, we were only able to design functioning primers for two of the eight candidate genes that were identified in RRBS analyses at the fledged juvenile stage and that had DMCs within the TSS or promoter. Nonetheless, using the results of our MS‐HRM analyses we tentatively suggest that perhaps 50% of the candidate genes that were identified in differential methylation analyses on the RRBS dataset may genuinely be involved in an epigenetic response to parasitism. This underscores the importance of exercising caution when constructing a narrative around putative candidate genes and highlights the value of performing follow‐up studies on candidate genes for traits of interest.

### 4.5 Caveats and future directions

Like many epigenetic studies in natural populations, we sampled blood, which comes with certain limitations (Husby, 2020, 2022). In our case, the use of whole blood can be considered a relevant tissue for studying methylation in relation to parasite infection because it contains white blood cells and other immune components (Scanes, 2015), and blood is useful because repeated sampling of the same individuals can easily be done. However, in birds red blood cells are nucleated (Scanes, 2015), so methylated DNA extracted from whole blood originates from a mixture of red and white blood cells alongside cell‐free DNA and other important immune components, as well as other cell types that can interfere with methylation estimates. As there is heterogeneity in the DNA methylation profiles of different blood cell types (Adalsteinsson et al., 2012), it is possible that the changes in methylation patterns that we observed in infected juvenile house sparrows are due to changes in the cell composition of whole blood during infection, rather than due to changes in DNA methylation levels in response to immune challenge per se (Husby, 2020, 2022). One solution to disentangle this is to examine methylation patterns in white blood cells only in future studies. However, with present methods large blood samples are needed to separate the buffy coat that contains the white blood cells and accounts for <1% of a whole blood sample, so repeated white blood cell sampling of small passerines is not currently feasible.

Because RRBS is a reduced representation technique, this study characterized only a limited number of the approximately 15 million CpG sites in a bird genome (Derks et al., 2016). Although RRBS data are enriched for CpG‐dense regulatory regions of the genome where DNA methylation changes are more likely to influence gene expression (Gu et al., 2011), it is possible that additional effects of infection by *S. trache*a on DNA methylation occurred in areas of the house sparrow genome that were not sequenced. Prior to differential methylation analyses, we removed CpG sites with less than 15% difference in mean methylation level between case and control birds because filtering out such sites is statistically convenient and relatively common (see e.g. Hu et al., 2018; Lindner, Verhagen, et al., 2021; Metzger & Schulte, 2018). However, the consequences of such filtering are not well understood and may lead to *p*‐value inflation and overestimation of the relative importance of DNA methylation on the phenotype (Husby, 2022). Thus, future developments of methods to control for the highly skewed *p*‐value distributions in differential methylation analyses without a priori filtering would hopefully mitigate this problem (see Husby, 2022 for more information). We were also limited by relatively low sample size in our RRBS analyses (*n* = 24 samples from *n* = 12 juvenile individuals, consisting of six cases and six controls that were sampled at both the nestling and fledged juvenile stage). Limited sample sizes are unfortunately a general problem in epigenetics studies on natural populations (Lea et al., 2017). For example, in Hu et al. (2018) three individuals per condition (case, control) were sampled at three timepoints, in Viitaniemi et al. (2019) eight individuals per condition (warm, cold) were sampled at four timepoints, and in McNew et al. (2021) nine individuals per condition (case, control) were sampled in 4 consecutive years. Nonetheless, the limited sample size of our RRBS dataset reduces the power to detect CpG sites with small differences in DNA methylation and the limited sample size could also lead to inflation of effect sizes. For the above reasons, it is important that the DMCs identified here should be regarded as candidate loci until verification or ideally functional studies have been done (Gudmunds et al., 2022). Accordingly, the MS‐HRM analyses we used to verify the relationship between methylation proportion and infection status for *NR1D1* and *CLDN22* serve as a first step toward substantiating the results of the differential methylation analyses in this study.

The present study compared naturally infected house sparrows to individuals that were defined as uninfected based on having several feces samples with zero *S. trachea* fecal egg count. Thus, this study is correlative in nature and potential misclassification of infected individuals as uninfected, as well as any differences in micro‐environment experienced by house sparrows living on the same island, could have influenced the results. Furthermore, as individual infection status in the present study was determined using FEC, it would have been interesting to examine whether methylation levels at all differentially methylated genes with immune‐related functions were correlated with FEC. However, FEC has some measurement error in the house sparrow because small fecal samples make standardization of FEC difficult (Holand et al., 2015), and irregular sampling of individuals along with the infection trajectory of *S. trachea* (Barus, 1966b) means that a large sample size is required to make inferences about the effect of FEC on any phenotype of interest. Thus, the relationship between methylation level and FEC could only be explored using the larger MS‐HRM dataset for *NR1D1* and *CLDN22*. Finally, because we did not measure RNA expression in our samples we cannot draw definite conclusions about the effects of DNA methylation differences on gene expression, although it has been demonstrated that DNA methylation in the TSS or promoter region of genes influences gene expression in an expected manner in the great tit (Laine et al., 2016) and in house sparrows (Lundregan et al., preprint), and that changes in DNA methylation can lead to changes in RNA expression (Lindner, Laine et al., 2021).

### 4.6 Conclusions

Parasites are major drivers of ecological and evolutionary processes in natural populations, and exert strong selective pressures on their hosts (Altizer et al., 2013; Morgan et al., 2004). There is increasing evidence that epigenetic modifications play an important role in mounting the immune response to parasite challenge. However, many previous studies used animal experiments (Cook et al., 2015; Hu et al., 2018; Sagonas et al., 2020), or experimental manipulation of parasite loads in natural populations (McNew et al., 2021), with some exceptions (see Berbel‐Filho et al., 2019; Wenzel & Piertney, 2014). Here, we demonstrate an epigenetic signature of naturally occurring parasite infection in the house sparrow, whereby mean genome‐wide methylation levels decreased in case birds upon infection by *S. trachea*, whereas mean genome‐wide methylation levels in control birds did not change over the same time period. Furthermore, we identified several DMCs in the TSS, promoter, or first intron of immune genes. These findings suggest that DNA methylation may play an additional role in genetic variation in allowing organisms to mount a plastic response to immune challenge by parasites in nature. Genes that were differentially methylated between cases and controls at the nestling stage were related to immune homeostasis and initial immune response, which may suggest that regulatory differences in these processes could make some birds more susceptible to parasite infection. Several genes that were differentially methylated between infected birds and controls at the fledged juvenile stage, as well as genes identified in the temporal differential methylation analysis, were related to innate and adaptive immune processes. Thus, parasite infection may result in DNA methylation changes at diverse immune genes. Nonetheless, in differential methylation analyses with limited sample size, and in genome scan studies more generally, caution should be exercised when constructing a narrative around identified genes (Pavlidis et al., 2012). Thus, genes close to the DMCs identified in the present study should be regarded as candidate genes until verified. As a first step toward this, we used MS‐HRM analyses and a larger dataset that included 322 fledged juvenile house sparrows from five islands in the metapopulation to verify the relationship between methylation proportion at *NR1D1* and *CLDN22* and *S. trachea* infection status. We found that methylation proportion at *NR1D1*, but not at *CLDN22*, remained related to infection status, which underscores the importance of performing follow‐up studies on candidate genes. The observed positive relationship between juvenile recruitment probability and methylation proportion at *NR1D1* in infected individuals suggests that birds may mount an epigenetic response to parasitism, which can result in a fitness advantage through increased survival probability. Taken together, the results of the present study highlight the potential for ecological epigenetics studies to provide a mechanistic understanding of host–parasite interactions in natural populations.

## AUTHOR CONTRIBUTIONS

**Sarah L. Lundregan:** Conceptualization (equal); data curation (lead); formal analysis (lead); methodology (equal); visualization (lead); writing – original draft (lead); writing – review and editing (equal). **Hannu Mäkinen:** Data curation (supporting); formal analysis (supporting); methodology (supporting); writing – review and editing (equal). **Amberly Buer:** Data curation (lead); formal analysis (supporting); investigation (supporting). **Håkon Holand:** Formal analysis (supporting); writing – review and editing (equal). **Henrik Jensen:** Conceptualization (equal); funding acquisition (equal); supervision (equal); writing – review and editing (equal). **Arild Husby:** Conceptualization (equal); funding acquisition (equal); methodology (supporting); supervision (equal); writing – review and editing (equal).

## CONFLICT OF INTEREST

The authors have no affiliations or associations, professional, financial, or otherwise, that could influence our objectivity.

## Data Availability

The phenotypic and epigenetic data used in this study is available on Dryad at https://doi.org/10.5061/dryad.h70rxwdnh.

## APPENDIX 1

**TABLE A1:** Number of raw reads in RRBS datasets, mapping efficiency and mean methylation percentage of unfiltered mapped reads for each sample (individual IDs are given after the underscore)

Sample	Sample timepoint	Raw reads	Mappability %	Methylated %
Juvenile dataset				
Case				
7_8N06323	Nestling	22,177,787	74.16	25.03
8_8N06323	Fledged juvenile	24,473,304	72.33	22.75
12_8N06545	Nestling	25,991,944	68.45	19.06
11_8N06545	Fledged juvenile	22,689,581	71.33	11.29
101_8M71818	Nestling	27,699,795	82.46	25.52
105_8M71818	Fledged juvenile	26,688,973	81.83	24.36
152_8M71882	Nestling	24,349,377	83.82	28.06
111_8M71882	Fledged Juvenile	27,007,381	82.32	23.54
158_8N06359	Nestling	23,279,380	83.64	25.74
9_8N06359	Fledged juvenile	24,842,452	73.19	18.12
159_8N06539	Nestling	26,680,875	84.05	20.02
10_8N06539	Fledged juvenile	23,517,181	69.82	20.24
Control				
13_8N06587	Nestling	24,334,733	71.18	22.72
137_8N06587	Fledged juvenile	25,876,269	81.95	22.77
22_8N06958	Nestling	23,179,413	68.60	13.89
119_8N06958	Fledged juvenile	23,348,719	82.67	27.09
102_8M71821	Nestling	24,554,948	81.59	24.88
108_8M71821	Fledged juvenile	27,515,560	83.82	13.32
103_8M71823	Nestling	24,417,496	83.11	26.67
109_8M71823	Fledged Juvenile	24,630,686	83.13	21.64
104_8M71828	Nestling	24,864,969	82.21	26.53
110_8M71828	Fledged juvenile	25,682,411	82.70	25.93
106_8M71819	Nestling	24,222,994	82.78	27.02
107_8M71819	Fledged juvenile	26,250,127	82.43	25.50
Adult dataset				
Case				
123_8309263	Infected	25,665,704	81.82	24.13
134_8816918	Infected	25,187,964	82.90	25.93
35_8L26591	Infected	25,354,002	68.33	22.03
84_8M31507	Infected	23,906,506	81.61	21.90
95_8M71205	Infected	25,066,404	81.06	24.80
Control				
136_8N06560	Never infected	26,461,600	84.00	20.28
60_8L89503	Never infected	23,277,949	71.15	21.70
42_8L19928	Never infected	23,041,669	70.92	23.60
117_8M72863	Never infected	37,064,847	79.87	24.63
61_8L89516	Never infected	26,480,484	81.63	29.64
	Mean:	25,287,750	78.73	22.95

**TABLE A2:** Number of 10× methylated sites in the juvenile and adult RRBS datasets

	No filtering	1×	3×	5×	10×
Nestlings (*n* = 12)					
CpG sites	2,627,193	1,436,550	1,105,563	874,911	393,138
Methylation % included	29.479	20.868	17.668	15.268	10.545
Methylation % excluded	‐	‐	31.557	26.772	19.122
Fledged juveniles (*n* = 12)					
CpG sites	2,654,038	1,668,582	1,335,539	1,101,818	619,626
Methylation % included	28.594	21.174	17.362	14.619	9.638
Methylation % excluded	‐	‐	36.461	30.293	21.020
Full juvenile dataset (*n* = 24)					
CpG sites	2,710,702	1,385,309	1,048,058	811,794	337,524
Methylation % included	28.920	19.446	15.652	12.844	8.014
Methylation % excluded	‐	‐	31.236	25.300	16.281
Adult dataset (*n* = 10)					
CpG sites	2,634,331	1,551,061	1,229,677	1,011,475	553,161
Methylation % included	28.726	22.851	20.604	18.766	14.179
Methylation % excluded	‐	‐	31.448	29.124	24.302

**TABLE A3:** *p*‐Values from comparison of mean methylation values between differing genomic regions (intergenic, intron, exon, promoter, TSS) using pairwise Wilcoxon rank sum tests

	Exon	Intergenic	First intron	Intron	Promoter
Intergenic	1.661e^−179^	‐	‐	‐	‐
First intron	6.498e^−66^	0.290	‐	‐	‐
Intron	1.203e^−84^	0.038	0.037	‐	‐
Promoter	<1e^−308^	7.581e^−148^	2.115e^−24^	1.324e^−075^	‐
TSS	<1e^−308^	<1e^−308^	9.792e^−187^	<1e^−308^	1.316e^−186^

*Note*: Methylation levels in intergenic regions and introns were similar, while methylation levels differed significantly between all other genomic regions.

**TABLE A4:** Significant sites in the TSS, promoter, or first intron of genes in differential methylation analyses on the RRBS data

Site position		Methylation % (case|control)	*p*‐value	Distance from gene (bp)	Genomic region	Gene	Gene function	References
Nestlings								
chr5	9,033,722	23.4|46.7	4.223 e^−9^	2172	Promoter	FNTB: Protein farnesyltransferase subunit beta	Protein farnesylation contributing to T‐cell homeostasis, T‐cell activation, and fate decisions	Du et al. (2020)
chr5	9,033,722	23.4|46.7	4.223 e^−9^	1671	Promoter	Rab15: Ras‐related protein Rab‐15	Targeted to Weibel‐Palade bodies, Rab15 is necessary for secretion of von Willebrand factor and P‐selectin. Exocytosis of WPB's releases P‐selectin to initiate leukocyte binding to endothelial cells. Rab GTPases modulate innate immune response	Nightingale and Cutler (2013), Valentijn et al. (2011) and Prashar et al. (2017)
chr5	7,759,764	46.7|75.9	4.800 e^−7^	246	TSS	Ssh3: Protein phosphatase Slingshot homolog 3	Activates Cofilin, a key regulator of Actin dynamics. Role in regulation of autophagy in macrophages. Downregulated in dendritic cells and monocytes	Pinto et al. (2021)
chrLGE22	123,412	14.1|39.0	5.217 e^−7^	2960	Promoter	Tmprss12: Transmembrane protease serine 12	Serine proteases are involved in many biological processes, including immune activation. Many helminth parasites have evolved serine protease inhibitors to alter the host's immune response	Molehin et al. (2012)
scaffold00435	52,109	30.9|2.9	2.543^−6^	446	Promoter	Myg1: UPF0160 protein MYG1 mitochondrial	Mitochondrial gene integral to oxidative phosphorylation. Dysregulation leads to increased ROS and oxidative stress, implicated in vitiligo	Grover et al. (2019)
chr19	4,588,448	39.1|16.3	3.074 e^−6^	1968	Promoter	Sebox: Homeobox protein SEBOX	Sebox is a maternal homeobox transcription factor that regulates degradation of mRNAs encoding many maternal factors and the expression of pluripotency genes	Zheng et al. (2013)
scaffold00561	10,513	38.9|17.3	1.038 e^−5^	1072	Promoter	WDR83OS: Protein Asterix	Component of the intermembrane chaperone PAT complex. Associates with divalent metal ion transporter TMD1	https://www.uniprot.org/uniprot/Q9Y284
Fledged juveniles								
chr23	3,077,582	12.8|41.8	6.415 e^−12^	392	Promoter	SF3A3: Splicing factor 3A subunit 3	The SF3A mRNA splicing complex controls production of a negative regulator of TLR signaling that limits the extent of innate immune activation	De Arras and Alper (2013)
chr14	7,040,962	10.4|42.1	8.445 e^−8^	‐	First intron	CCZ1: Vacuolar fusion protein CCZ1 homolog	Enables guanyl‐nucleotide exchange factor activity. Predicted to be involved in vesicle‐mediated transport. Located in intracellular membrane‐bounded organelles	https://www.ncbi.nlm.nih.gov/gene/51622
chr25	394,434	28.1|53.4	3.187 e^−7^	‐	First intron	RAB25: Ras‐related protein Rab‐25	The protein encoded by this gene is a member of the RAS superfamily of small GTPases. The encoded protein is involved in membrane trafficking and cell survival	https://www.ncbi.nlm.nih.gov/gene/57111
chr13	1,914,936	30.2|56.4	3.235 e^−7^	1247	Promoter	Rell2: RELT‐like protein 2	Rell2 is a homologous binding partner of RELT. RELT is primarily expressed in immune cells and modulates immunity by co‐stimulating T‐cell production, and contributing to CD4+ cell homeostasis	Sica et al. (2001) and Choi et al. (2018)
chr10	15,247,962	63.3|41.93	1.173 e^−6^	616	Promoter	RGMA: Repulsive guidance molecule A	RGMA is a GPI‐anchored glycoprotein that induces CD4+ T‐cell activation and increases immune response	Fujita and Yamashita (2017)
chr8	44,899,349	20.7|57.2	1.208 e^−6^	485	Promoter	SH3BGRL: SH3 domain‐binding glutamic acid‐rich‐like protein	Downregulated in cells transformed by avian retrovirus v‐rel oncogene (v‐rel binds promoter)	Majid et al. (2006)
scaffold00317	199,582	85.2|64.1	2.256 e^−6^	404	Promoter	GBA: Glucosylceramidase	Knockout of GBA leads to widespread dysfunction of immune cells. T‐cell maturation, B‐cell recruitment, antigen presentation	Liu et al. (2012)
chr27	3,343,974	64.4|36.3	2.547 e^−6^	671	Promoter	NR1D1: Nuclear receptor subfamily 1 group D member 1	Regulates development of Th17 cells. Represses TLR4, IL‐6, and Cx3cr1 expression in macrophages. NR1D1 silencing activates MAPK and NF‐κβ. Regulates ROS generation	Liu et al. (2020)
chr22	2,776,372	19.3|43.2	2.786 e^−6^	‐	First intron	STC1: Stanniocalcin‐1	Elevated expression in colon of humans infected by the protozoan parasite Entamoeba histolytica. Early response to mechanical injury of epithelial cells. Wound closure	Zhang and Stanley Jr (2004)
scaffold00304	44,417	44.0|72.3	4.175 e^−6^	148	TSS	TRIM54: Tripartite motif‐containing protein 54	TRIM proteins have diverse functions in innate immunity. Induced by type I and II interferon, which are crucial for resistance to pathogens. Regulation of TLR signaling. Regulate signaling molecules downstream of PRRs	Jefferies et al. (2011)
chr24	266,363	33.3|9.1	4.468 e^−6^	107	TSS	CLDN22: Claudin‐22	Claudins alter epithelial permeability by regulating tight junction permeability. Helminth infection has been linked to increase in epithelial permeability and tight junctional changes in pulmonary cells	Su et al. (2011) and Soini (2011)
Temporal methylation change								
chr11	13,246,201	−12.2|14.4	9.878 e^−10^	2241	Promoter	TPPP3: Tubulin polymerization‐promoting protein family member 3	TPPP3 expression reduced in carcinomas. Negatively correlated with CD8+ T‐cell and memory B cell expression	Yang et al. (2020)
chr18	1,077,705	−75.4|−43.9	5.863 e^−9^	2597	Promoter	Bahcc1: BAH and coiled‐coil domain‐containing protein 1	Recognizes trimethylated histone H3 lysine 27 and enforces silencing of demarcated genes. Highly expressed in acute leukemia	Fan et al. (2020)
chr2	116,184,130, 116,184,119	−13.2|10.1	8.606 e^−9^	320	Promoter	CYC: Cytochrome c	Mitochondrial DAMP, recognized by PRRs of immune cells. Initiates apoptosis in pathogen infection	Eleftheriadis et al. (2016) and Song et al. (2020)
chr5	10,091,473	−29.2|25.3	8.865 e^−9^	691	Promoter	AKIP1: A‐kinase‐interacting protein 1	Regulates the rate of NF‐kappa β nuclear translocation, NF‐kappa β is a known regulator of immunity and inflammation	King et al. (2011)
chr23	6,147,354	−15.7|9.6	2.087 e^−8^	237	TSS	Tceb3: Transcription elongation factor B polypeptide 3	General transcriptional elongation factor for RNA polymerase II	Aso et al. (1995)
scaffold00561	3298	−18.5|12.1	3.173 e^−8^	857	Promoter	Dhps: Deoxyhypusine synthase	Governs mRNA translation and promotes post‐transcriptional regulation of inflammatory signaling in macrophages. Dhps also governs macrophage migration to sites of injury	Anderson‐Baucum et al. (2021)
chr18	468,736	8.7|−21.8	3.362 e^−8^	2065	Promoter	CANT1: Soluble calcium‐activated nucleotidase 1	Apyrase that prevents accumulation of UDP and maintains flux of UDP‐glucose. Important for maintaining glycoprotein synthesis. UDP‐glucose triggers innate mucosal immunity by inducing IL8 production	Arase et al. (2009)
chr18	8,686,738	−8.9|17.5	4.199 e^−8^	1801	Promoter	Uts2r: Urotensin‐2 receptor	Receptor for urotensin 2, urotensin 2 expression has been positively associated with helminth egg count in humans	Lee et al. (2019)
chr3	43,310,351	−7.8|12.2	2.866 e^−6^	‐	First intron	TARBP1: Probable methyltransferase TARBP1	Methyltransferase activity	Wu et al. (2008)
chr21	2,397,615, 2,397,622	−4.2|18.3	1.282 e^−7^	2219	Promoter	PRDM2: PR domain zinc finger protein 2	PRDM2 is recruited to damaged chromatin sites leading to H3K9 methylation, which is associated with transcriptional repression	Khurana et al. (2014)
chr13	2,270,178	18.5|−9.3	3.258 e^−6^	‐	First intron	APBB3: Amyloid beta A4 precursor protein‐binding family B member 3	The protein encoded by this gene is a member of the APBB protein family. It is found in the cytoplasm and binds to the intracellular domain of the Alzheimer's disease beta‐amyloid precursor protein (APP) as well as to other APP‐like proteins	https://www.ncbi.nlm.nih.gov/gene/10307
chr12	7,803,840	−40.4|−2.9	1.403 e^−7^	571	Promoter	Abhd6: Monoacylglycerol lipase ABHD6	Transmembrane serine hydrolase hydrolyses fatty acid monoacylglycerols. Abhd6 is a CB1 ligand highly expressed in the immune system. Inhibition of CB1 receptors results in increased egg count in helminth infection	Malamas et al. (2021) and Batugedara et al. (2018)
chr1A	54,089,745	−17.4|19.6	1.464 e^−7^	86	TSS	A4GALT: Lactosylceramide 4‐alpha‐galactosyltransferase	A4GALT deficient mice lack globo‐series glycosphingolipids that attenuate toxicity of LPS by binding to TLR4‐MD2	Kondo et al. (2013) and Zakeri et al. (2018)
chr17	6,612,566	−11.6|21.4	2.570 e^−7^	1485	Promoter	NUP214: Nuclear pore complex protein Nup214	Critical role in nucleocytoplasmic transport	https://www.uniprot.org/uniprot/P35658
chr25	388,799	−15.7|13.1	3.741 e^−7^	2578	Promoter	RAB11A: Ras‐related protein Rab‐11A	Rab11A controls TLR4 transport and IRF3 activation	Husebye et al. (2010)
chr28	1,448,200	−7.3|14.3	5.280 e^−7^	1065	Promoter	RORB: Nuclear receptor ROR‐beta	IL17 expression, Th17 differentiation	Okada et al. (2017)
chr15	2,135,684	−26.5|6.3	6.693 e^−7^	1286	Promoter	VPREB3: Pre‐B lymphocyte protein 3	Binding of free immunoglobulin light chains to VPREB3 inhibits their secretion and maturation in chicken B‐cells	Rosnet et al. (2004)
chr18	5,878,889	13.1|−16.8	6.763 e^−7^	2215	Promoter	Nucleoside diphosphate kinase	Regulation of mitochondrial respiration, apoptotic signaling	Schlattner et al. (2009)
chr28	830,179	−19.0|7.1	9.130 e^−7^	2713	Promoter	Hmg20b: SWI/SNF‐related matrix‐associated Actin‐dependent regulator of chromatin subfamily E member 1‐related	Synonym BRAF35, histone demethylase. Plays a role in removal of H3K9me2 marks, a prerequisite for transcription factor binding and induction of genes during PRR activation	Zhang and Cao (2019) and Kooistra and Helin (2012)
chr28	3,159,162	−20.0|14.6	9.859 e^−7^	1763	Promoter	med16: Mediator of RNA polymerase II transcription subunit 16	Coactivators of lipopolysaccharide and heat‐shock‐induced transcriptional activators	Kim et al. (2004)
chr9	25,075,683	−14.0|3.6	1.158 e^−6^	2653	Promoter	CLSTN2: Calsyntenin‐2	Learning, memory	https://www.ncbi.nlm.nih.gov/gene/64084
chr19	4,720,519	−6.4|10.7	1.423 e^−6^	2538	Promoter	HIC1: Hypermethylated in cancer 1 protein	All‐trans retinoic acid‐dependent transcription factor required for innate lymphoid cell homeostasis. ILCs are regulators of immune response at barrier sites such as the intestine	Burrows et al. (2018)
chr12	10,712,551	13.0|−13.9	1.579 e^−6^	168	TSS	KLHDC8B: Kelch domain‐containing protein 8B	Required for mitotic integrity and maintenance of chromosomal stability	https://www.uniprot.org/uniprot/Q8IXV7
chr11	11,826,529	−1.4|−19.4	1.592 e^−6^	1365	Promoter	TRADD: Tumor necrosis factor receptor type 1‐associated DEATH domain protein	Mediates antiviral immunity, increased expression in response to LPS	Li et al. (2021)
chr23	3,751,679	9.3|−17.9	1.745 e^−6^	667	Promoter	LSM10: U7 snRNA‐associated Sm‐like protein Lsm10	Formation of mature histone mRNA, important for epigenetic inheritance of gene expression patterns	Marzluff et al. (2008)
chr13	11,628,157	−24.9|9.6	2.238 e^−6^	2384	Promoter	Gm2a: Ganglioside GM2 activator	Known inflammatory regulator binds platelet‐activating factor PAF which promotes production of ROS. Contains ML domain involved in innate immunity	Inohara and Nuñez (2002) and Borges et al. (2017)
scaffold00314	126,473	−16.4|9.4	2.336 e^−6^	1236	Promoter	ANO8: Anoctamin‐8	Assembly of Ca2+ signaling complexes	Jha et al. (2019)
chr10	20,096,709	−18.6|4.9	2.621 e^−6^	268	TSS	KIF23: Kinesin‐like protein KIF23	Mitotic spindle processing drives microtuble movement	https://www.ncbi.nlm.nih.gov/gene/9493
chr18	1,101,328	−8.7|13.6	3.545 e^−6^	9	TSS	FSCN2: Fascin‐2	Actin crosslinkage, photoreceptor disk morphogenesis	https://www.ncbi.nlm.nih.gov/gene/25794
scaffold00282	89,697	−13.0|9.5	3.784 e^−6^	134	TSS	foxj1.2: Forkhead box protein J1.2	Motile ciliary function, clearance of mucus in lungs	Wallmeier et al. (2019)
chr23	3,901,868	−14.7|14.1	4.100 e^−6^	2044	Promoter	TEKT2: Tektin‐2	Structural component of ciliary and flagellar microtubules	https://www.uniprot.org/uniprot/Q9UIF3
chr18	10,888,389	14.5|−14.0	4.570 e^−6^	1469	Promoter	NTN1: Netrin‐1	Immunomodulatory function through regulation of leukocyte migration. Downregulated during lung infection during mice, which allows a rapid influx of inflammatory leukocytes to fight infection	Ly et al. (2005)
scaffold00950	10,033	−16.6|9.8	4.821 e^−6^	734	Promoter	MIOX: Inositol oxygenase	Degrades myo‐inositol that is important in metabolism. High expression of MIOX is related to markers indicating metabolic stress, including production of ROS	Gonzalez‐Uarquin et al. (2021)
scaffold00224	141,235	−7.8|19.9	6.666 e^−6^	2080	Promoter	S100A16: Protein S100‐A16	Involved in cancer progression	Tu et al. (2021)
chr18	11,102,130	10.5|−21.1	6.706 e^−6^	193	TSS	CYP2C5: Cytochrome P450 2C5	Catalyzes the 21‐hydroxylation of progesterone, resulting in the formation of deoxycorticosterone	https://www.uniprot.org/uniprot/P00179
scaffold00310	30,563	−8.9|19.1	6.770 e^−6^	2611	Promoter	USP2: Ubiquitin carboxyl‐terminal hydrolase 2	Prevents inappropriate activation of Imd‐dependent immune signaling by promoting Imd proteasomal degradation	Engel et al. (2014)
scaffold00683	33,800	−18.3|7.7	6.793 e^−6^	‐	First intron	DPYSL5: Dihydropyrimidinase‐related protein 5	This gene encodes a member of the CRMP (collapsing response mediator protein) family thought to be involved in neural development	https://www.ncbi.nlm.nih.gov/gene/56896
chr1A	47,819,230	−11.6|13.7	7.947 e^−6^	502	Promoter	GCAT: 2‐amino‐3‐ketobutyrate coenzyme A ligase mitochondrial	Phosphate‐dependent aminotransferase. Degradation of L‐theanine to glycine	https://www.ncbi.nlm.nih.gov/gene/23464
chr21	4,142,535	11.9|−19.5	7.970 e^−6^	13	TSS	DDX19B: ATP‐dependent RNA helicase DDX19B	Suppresses IFN production by promoting degradation of TBK1 and IKKε. Type I IFNs are upregulated during induction of the innate immune response	Chathuranga et al. (2021)
chr23	5,767,735	−10.3|9.9	8.442 e^−6^	1949	Promoter	syf2: Pre‐mRNA‐splicing factor syf2	Cell cycle regulator at G1/S transition	https://www.ncbi.nlm.nih.gov/gene/25949
scaffold00340	137,774	14.6|−14.9	8.684 e^−6^	782	Promoter	efna5b: Ephrin‐A5b	Region‐specific apoptosis during early brain development	Park et al. (2013)

*Note*: Site position, methylation percentage in cases vs. controls, *p*‐value, genomic region, gene, and any known gene functions are given.

**TABLE A5:** Results from GO functional analyses performed separately on the sets of candidate genes (genes with significantly differentially methylated sites in the TSS, promoter, or first intron) from differential methylation analyses in nestlings and fledged juveniles, as well on temporal methylation change

ID	Term	Ontology source	Bonferroni corrected *p*‐value	GO group	% associated genes	Nr. Genes	Associated genes
Nestlings							
No enriched functional terms							
Fledged juveniles							
GO:0003382	Epithelial cell morphogenesis	GO_BiologicalProcess‐EBI‐UniProt‐GOA‐ACAP‐ARAP_13.05.2021_00h00	1.6e^−4^	1	5.5	2	RAB25, STC1
Temporal methylation change							
R‐HSA:5357786	TNFR1‐induced proapoptotic signaling	REACTOME_Pathways_13.05.2021	.001	1	15.4	2	TRADD, USP2
GO:0001754	Eye photoreceptor cell differentiation	GO_BiologicalProcess‐EBI‐UniProt‐GOA‐ACAP‐ARAP_13.05.2021_00h00	.003	2	4.1	2	FSCN2, RORB
GO:0045773	Positive regulation of axon extension	GO_BiologicalProcess‐EBI‐UniProt‐GOA‐ACAP‐ARAP_13.05.2021_00h00	.004	3	4.3	2	NTN1, RAB11A
R‐HSA:5357956	TNFR1‐induced NF‐kappa B signaling pathway	REACTOME_Pathways_13.05.2021	.006	1	6.7	2	TRADD, USP2
GO:0042462	Eye photoreceptor cell development	GO_BiologicalProcess‐EBI‐UniProt‐GOA‐ACAP‐ARAP_13.05.2021_00h00	.006	2	5.4	2	FSCN2, RORB
R‐HSA:75893	TNF signaling	REACTOME_Pathways_13.05.2021	.007	1	4.5	2	TRADD, USP2
R‐HSA:5357905	Regulation of TNFR1 signaling	REACTOME_Pathways_13.05.2021	.007	1	5.7	2	TRADD, USP2

*Note*: Functional analysis was carried out using the Cytoscape plugin ClueGO 2.5.8, using Bonferroni step‐down correction with a cutoff *p*‐value of .05, default Kappa score, and default network specificity. Human gene ontologies were used and terms from GO biological process, KEGG, and Reactome pathways were considered.

**TABLE A6:** Primer information, amplified product information, and PCR conditions used in MS‐HRM

Gene	Primers	Length (bp) of product	n(CpGs)	PCR conditions	GenBank sequence ID: Position
NR1D1	F: TGGTGTTGTTGAGTATTTTTTTG R: CCCTACTCCTTCTAAAACTCCC	111	9	95°C (10 min), [95°C (15 s); 64°C (1 min); AutoDelta step‐down to 55°C]*55 cycles	CM004553.1 (chr 27): 3343927–3344037
CLDN22	F: GGGGTTGTTGGGTAAAGG R: CCCCCTCCTCCTAAATAATACA	174	6	95°C (10 min), [95°C (15 s); 60°C (1 min)]*60 cycles	CM004550.1 (chr 24): 266438–266611

*Note*: Optimal PCR conditions for NR1D1 that gave maximum PCR product amplification required use of AutoDelta for gradual step‐down of the annealing temperature from 64 to 55°C. optimal PCR conditions for CLDN22 did not require use of AutoDelta. The GenBank ID and sequence position are given for each amplicon.

**TABLE A7:** Results of generalized linear mixed‐effects models using the methylation‐sensitive high‐resolution melting (MS‐HRM) dataset to investigate the relationship between methylation proportion at NR1D1 and CLDN22 and age in days

Gene	Infection status	Age	Relmat.	ID	Year	Island	Residual
NR1D1	0.021 (.567)	0.001 (.165)	0.017 (0.127)	0.009 (0.093)	0.011 (0.106)	0.002 (0.047)	0.022 (0.147)
CLDN22	−0.005 (.644)	0.0004 (.023)	0.003 (0.054)	0.000 (0.000)	0.000 (0.013)	0.000 (0.016)	0.000 (0.011)

*Note*: Models were run using the subset of individuals for which information on age in days was available (*n* = 130 individuals for NR1D1 and *n* = 124 individuals for CLDN22). Effect size (above) and *p*‐value (below in parenthesis) are given for the main effects of infection status and age in days on methylation proportion. Variance (above) and standard deviation (below in parenthesis) are given for the random factors pedigree relatedness (Relmat), individual ID, year, island, and the residual variance.
